# Listening for extinction: Range‐wide occupancy highlights critical risks and priorities for northern spotted owls

**DOI:** 10.1002/eap.70294

**Published:** 2026-08-22

**Authors:** Damon B. Lesmeister, Julianna M. A. Jenkins, Raymond J. Davis, Natalie M. Rugg, Cara L. Appel, Larissa L. Bailey, Erin Bayne, Tara Chestnut, Leila S. Duchac, Katie M. Dugger, Taylor D. Ellis, Alan B. Franklin, Scott A. Gremel, Aaron Henderson, Edward Henson, J. Mark Higley, James E. Hines, Bruce Hollen, William L. Kendall, Taal Levi, Darryl I. MacKenzie, Christopher E. McCafferty, Thomas Munger, David T. Press, Caroline Provost, Suzanne Reffler, Zachary J. Ruff, James K. Swingle, Erica Tevini, Alaina D. Thomas, Matthew J. Weldy, Kirsten Wert, Angela White, Charles B. Yackulic

**Affiliations:** ^1^ U.S. Forest Service, Pacific Northwest Research Station Corvallis Oregon USA; ^2^ Department of Fisheries, Wildlife, and Conservation Sciences Oregon State University Corvallis Oregon USA; ^3^ U.S. Forest Service, Pacific Northwest Region Corvallis Oregon USA; ^4^ Department of Fish, Wildlife, and Conservation Biology Colorado State University Fort Collins Colorado USA; ^5^ Department of Biological Sciences University of Alberta Edmonton Alberta Canada; ^6^ U.S. National Park Service, Mount Rainier National Park Ashford Washington USA; ^7^ Department of Forest, Rangeland, and Fire Sciences University of Idaho Moscow Idaho USA; ^8^ U.S. Geological Survey, Oregon Cooperative Fish and Wildlife Research Unit, Department of Fisheries, Wildlife, and Conservation Sciences Oregon State University Corvallis Oregon USA; ^9^ U.S. National Park Service, Point Reyes National Seashore Point Reyes Station California USA; ^10^ U.S. Department of Agriculture Wildlife Services, National Wildlife Research Center Fort Collins Colorado USA; ^11^ U.S. National Park Service, Olympic National Park Port Angeles Washington USA; ^12^ Hoopa Valley Tribal Council, Forestry Division Hoopa California USA; ^13^ U.S. Geological Survey, Patuxent Wildlife Research Center Laurel Maryland USA; ^14^ Bureau of Land Management, Oregon State Office Portland Oregon USA; ^15^ U.S. Geological Survey, Colorado Cooperative Fish and Wildlife Research Unit Colorado State University Fort Collins Colorado USA; ^16^ Proteus Research and Consulting Ltd Outram New Zealand; ^17^ California State Parks, Sonoma‐Mendocino Coast District Mendocino California USA; ^18^ Forest Ecosystems and Society, Oregon State University Corvallis Oregon USA; ^19^ U.S. Forest Service, Pacific Southwest Research Station Davis California USA; ^20^ U.S. Geological Survey, Southwest Biological Science Center Flagstaff Arizona USA

**Keywords:** barred owl, bioacoustics, convolutional neural networks, extinction vortex, Northwest Forest Plan, occupancy modeling

## Abstract

The northern spotted owl (*Strix occidentalis caurina*) continues to decline across its range, threatened by habitat disturbances and invasive barred owls (*Strix varia*). We conducted a range‐wide spotted owl occupancy assessment using 2.1 million h of passive acoustic recordings from 4081 survey stations within 1027 randomly selected 5‐km^2^ hexagons across six physiographic regions. Applying multistate occupancy models, we estimated probabilities of landscape use (by at least one northern spotted owl) and pair occupancy (occupied by both a male and a female), while accounting for imperfect and sex‐biased detection probability. Detection probability of pairs was low in most regions, primarily due to undetected females, but increased with survey effort. If management decisions can only be made when there is a high probability of detecting both sexes when they are present, then longer surveys are one approach to support the decisions. Landscape use and pair occupancy were positively associated with old‐growth forest structure and topographic diversity. We also found a pronounced latitudinal gradient in spotted owl landscape use rates with estimated mean landscape use of 0.17 (SE = 0.03) and 0.16 (SE = 0.02) in the two most northern regions, and 0.54 (SE = 0.04) and 0.52 (SE = 0.05) in the two most southern regions. Pair occupancy rates were higher in southern regions (0.29, SE = 0.03, 0.43, SE = 0.05) and very low in northern regions (0.04, SE = 0.01, 0.03, SE = 0.01). In contrast to spotted owls, barred owls were detected in 90% of northern hexagons and 50% of southern hexagons, and barred owl calling intensity was over eight times higher than spotted owls. These results highlight extinction risks in several regions and suggest that management actions, such as spatially targeted barred owl control and protection of structurally complex habitats, have a narrow temporal window in which they can be effective in lowering the probability of regional extirpations. We generated predictive maps of landscape use and pair occupancy, which could help guide regional conservation strategies. This study demonstrates how passive acoustic monitoring and machine learning can be used for broad‐scale ecological monitoring, identifying extinction risk thresholds, supporting adaptive management, and improving conservation outcomes for wide‐ranging and elusive species that can reliably be detected through unique vocalizations.

## INTRODUCTION

Ecological communities are increasingly disrupted by human‐induced changes, including alterations to habitat quality, invasive species, and altered disturbance regimes, leading to declines in biodiversity and ecosystem resilience worldwide (Cardinale et al., 2012; Dirzo et al., 2014). The management responses that mitigate these impacts are often more effective if based on robust ecological monitoring programs that accurately and efficiently determine species' status across large spatial and temporal scales. Our ability to detect emerging conservation crises is limited because monitoring methods often fall short in efficiency, scale, and sensitivity (Nichols & Williams, 2006). Noninvasive monitoring methods and advancements in remote sensing technologies are improving the ability to effectively monitor populations at large scales (Bush et al., 2017; Tosa et al., 2021).

The northern spotted owl (*Strix occidentalis caurina*) exemplifies the challenges faced by many species dependent on mature, structurally complex Douglas fir (*Pseudotsuga menziesii*) and mixed conifer forests (Forsman et al., 1984; Sovern et al., 2019). Once widespread across late‐successional forests of the Pacific Northwest, the northern spotted owl experienced substantial range‐wide population declines during the 20th century due to extensive habitat loss from logging, prompting its listing as threatened under the U.S. Endangered Species Act in 1990 (USFWS, 1990) and timber harvest injunctions (Hungerford, 1994). In response, the US federal land management agencies implemented the Northwest Forest Plan (NWFP) in 1994 to protect and restore late‐successional forests on federal lands, shifting forest management priorities toward biodiversity conservation (Spies et al., 2019).

Despite these conservation measures on federally managed forests, population recovery has not occurred for several avian species associated with structurally complex old forest, suggesting additional mechanisms are drivers of contemporary population trends (Phalan et al., 2019). Northern spotted owl populations continue to decline faster than expected if habitat loss alone were driving population change (Yackulic et al., 2019), and by 2020 were found to warrant uplisting to endangered status (USFWS, 2020). Concern is particularly high that some areas of Washington and Oregon are approaching functional extinction thresholds. As of 2017, pair occupancy of monitored territories in some areas had fallen below 0.20 (Franklin et al., 2021), which is a level associated with high risk of demographic collapse (Noon & McKelvey, 1996). These population declines are likely driven primarily through competition with invasive barred owls (*S. varia*), with continuing habitat loss (e.g., timber harvest, wildfire), legacy effects from historical logging, and altered disturbance regimes also contributing (Franklin et al., 2021). Barred owl is a species that recently expanded from north to south across the northern spotted owl geographic range (Lesmeister et al., 2018; Wiens et al., 2021). Barred owls outcompete northern spotted owls for resources such as nesting sites and prey, excluding the native owl from breeding sites, and ultimately decrease key vital rates like survival that drive population declines (Franklin et al., 2021; Jenkins et al., 2021; Jenkins, Lesmeister, Forsman, et al., 2019; Wiens et al., 2014; Yackulic et al., 2019).

The first phase of northern spotted owl population monitoring under the NWFP was achieved primarily through demographic studies utilizing mark‐resight methods (Lint et al., 1999), which involved capturing, marking, and repeatedly observing individuals over multiple years on 11 study areas (Franklin et al., 2021). These data provided baseline demographic rates, such as survival, reproduction, recruitment, and rates of population change. Estimates of these demographic rates have allowed researchers to identify the relative importance of different factors associated with continued population declines. The success of this approach has served as a reference when evaluating newer, less‐invasive monitoring techniques. Persistent declines in northern spotted owl populations in some of the study areas resulted in increasing uncertainty in estimates of vital rates (Dugger et al., 2016), which led to explorations of alternative monitoring methods. Passive acoustic monitoring, coupled with machine learning techniques, has emerged as an alternative for northern spotted owl monitoring that performs well (Duchac et al., 2020; Lesmeister & Jenkins, 2022; Ruff et al., 2021). Passive acoustic monitoring allows for extensive, continuous, noninvasive collection of detection/non‐detection data, enhancing our ability to monitor cryptic and rare species, detect ecosystem change, and inform conservation actions.

Here, we present a multistate occupancy assessment of northern spotted owls based on passive acoustic monitoring data collected throughout their geographic range in the United States. Our objectives were to quantify detection probabilities, identify environmental factors influencing northern spotted owl distribution, and assess regions nearing or crossing extinction thresholds. We define and estimate two complementary occupancy state variables at the same spatial scale (5‐km^2^ hexagons): landscape use and pair occupancy. Landscape use refers to the probability that a hexagon was used by at least one territorial northern spotted owl (of any sex) during the 6–8‐week survey period. This metric captures broader space‐use patterns, including transient individuals or intermittent calling behavior, and is particularly relevant for wide‐ranging species where the assumption of strict site closure is unlikely to hold (Bailey et al., 2014; Kendall et al., 2013; Rota et al., 2009). In contrast, pair occupancy is the conditional probability that a hexagon used by northern spotted owls was occupied by both a territorial male and a female, a state spatially restricted and indicative of potential reproduction and therefore linked to population viability. Together, these metrics provide a nuanced understanding of species status: landscape use informs connectivity and distribution, while pair occupancy is associated with potential demographic health and extinction risk. Regions with low pair occupancy, even where landscape use persists, may represent functional population collapse with a narrow temporal window existing for conservation interventions to reverse declines.

While we did not conduct formal extinction threshold analyses, we interpret areas with very low pair occupancy rates, particularly those falling below 0.20, as approaching functional extinction, based on thresholds proposed in prior demographic and viability studies (Noon & McKelvey, 1996). These inferences are grounded in comparative modeling and empirical evidence and can serve to identify regions where conservation urgency is highest even in the absence of formal extinction probability modeling. We did not intend to generate a habitat model, but rather to create a predictive model of northern spotted owl distribution in 2023 and to quantify the degree to which current distribution aligns with models of nesting and roosting forest, a critical component of northern spotted owl habitat (Davis et al., 2022; Lesmeister et al., 2018).

## METHODS

### Study area

**FIGURE 1:**
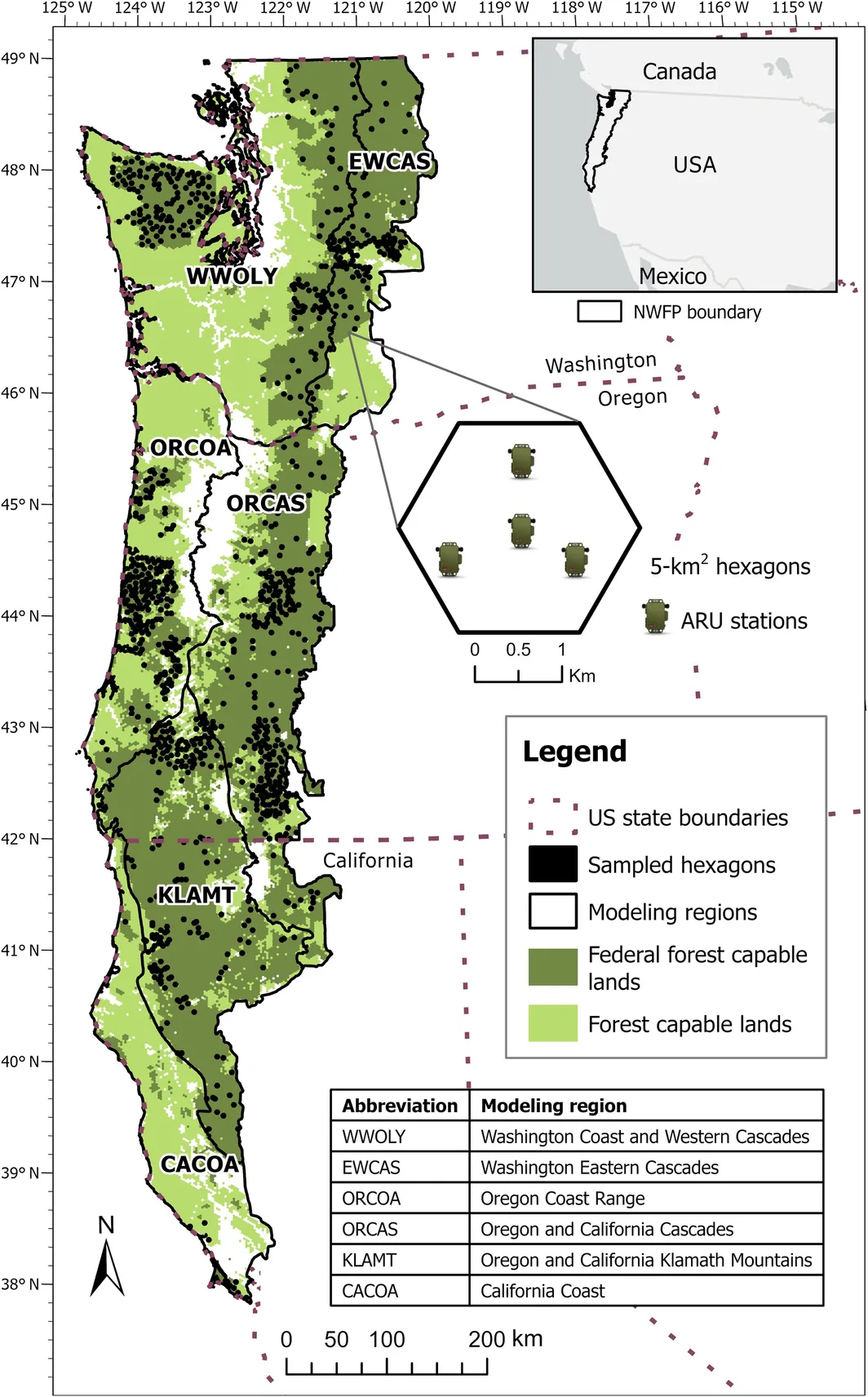
Sampling frame of hexagons used in passive acoustic monitoring of northern spotted owls and barred owls across the range of the northern spotted owl in the Pacific Northwest, USA. Six modeling regions were delineated based on ecological similarities of the physiographic provinces. The dark green area is where the pool of 5‐km^2^ hexagons that are ≥50% forest capable and ≥25% under federal land management are located. The light green areas represent where hexagons that are ≥50% forest capable under nonfederal management are located and black outlines are modeling region boundaries within the Northwest Forest Plan (NWFP) boundary. The black points are 5‐km^2^ hexagons that were randomly selected for passive acoustic monitoring from all dark green hexagons using the 2 + 20% design (Lesmeister, Appel, et al., 2021). Within each selected hexagon, we deployed four autonomous recording units (ARUs; figure inset) for a 6–8‐week survey period.

We collected data within the NWFP area on 15 National Forests, five Bureau of Land Management districts, and nine National Park units that were under federal management. The federal lands encompassed by the NWFP were ecologically diverse and covered approximately 98,740 km^2^ across western Washington, western Oregon, and northwestern California, aligning closely with the geographic range of the northern spotted owl (Figure 1). We also included data collected on the Hoopa Valley Reservation and California State Parks to bolster samples from northwestern California. The lands within the NFWP area support a diversity of forest ecosystems, notably Douglas fir forests, which dominate the western slopes of the Cascade Range and coastal regions, commonly interspersed with species such as western hemlock (*Tsuga heterophylla*) and western red cedar (*Thuja plicata*). Mixed‐conifer forests, which are prevalent in the Klamath Mountains and eastern portions of the Cascades, were characterized by a mix of Douglas fir, grand fir (*Abies grandis*), white fir (*Abies concolor*), incense cedar (*Calocedrus decurrens*), sugar pine (*Pinus lambertiana*), and ponderosa pine (*Pinus ponderosa*). The Klamath Mountains were unique with early seral stages that were commonly dominated by hardwood and transitioning to older stands dominated by conifer species with midstories of hardwood species, such as tanoak (*Notholithocarpus densiflorus*) and Pacific madrone (*Arbutus menziesii*) (Franklin et al., 2000). Coastal California was dominated by coast redwood (*Sequoia sempervirens*) forests transitioning to the mixed‐conifer forests of the Klamath Mountains inland.

Elevations across the NWFP landscape vary dramatically, ranging from sea level along coastal regions to over 2500 m in mountainous terrain. Correspondingly, climate conditions are highly variable, with cool, wet winters and hot, dry summers at lower elevations, transitioning to colder temperatures and substantial snowfall at higher elevations. Annual precipitation is equally variable, averaging 75 cm in drier, interior regions and exceeding 250 cm in coastal and mountainous areas (Forsman et al., 2011).

Our range of sampling included 11 study areas with long‐term northern spotted owl population monitoring. Ten of the study areas (Olympic Peninsula, Cle Elum, Mount Rainier National Park, Oregon Coast Range, HJ Andrews Experimental Forest, Tyee, Klamath, Oregon South Cascades, Hoopa Valley Tribe Reservation, Northwest California) were long‐term demographic study areas for northern spotted owl monitoring under the NWFP (Franklin et al., 2021). Our sampling also covered an additional long‐term study area (Marin County) that had not been included in previous demographic monitoring meta‐analyses.

### Data collection

We created a tessellated layer of 5‐km^2^ hexagon sampling units that covered the entire historical range of the northern spotted owl, excluding British Columbia, Canada (Lesmeister, Appel, et al., 2021). We delineated the study area into six modeling regions by combining physiographic provinces based on ecological similarity (Davis et al., 2022): Oregon and California Klamath Mountains (KLAMT; *n* = 191 hexagons), California Coast (CACOA; *n* = 11), Oregon and California Cascades (ORCAS; *n* = 300), Oregon Coast Range (ORCOA; *n* = 208), Washington Coast and Western Cascades (WWOLY; *n* = 212), and Washington Eastern Cascades (EWCAS; *n* = 105; Figure 1). The hexagon size we used was approximately the size of a northern spotted owl territory core area (Glenn et al., 2004; Schilling et al., 2013) and approximated the home range size reported for barred owls in the Pacific Northwest (Hamer et al., 2007; Wiens et al., 2014). We selected sampling units using a 2 + 20% design, in which we randomly selected 2% of available 5‐km^2^ hexagons throughout the study area, stratified by physiographic province, and 20% of hexagons within demographic study areas (Lesmeister & Jenkins, 2022; Lesmeister, Appel, et al., 2021). We considered hexagons as available if they contained ≥50% forest capable lands and ≥25% federal ownership and were safely accessible by field crews (Figure 1). Forest capable lands were those areas environmentally suitable for developing into any type of forest (Davis et al., 2011).

Within each hexagon, we deployed four autonomous recording units (ARUs) as our sampling stations for a 6–8‐week survey period (binned in 1‐week survey occasions) between February 24 and September 6, 2023, which corresponded to the northern spotted owl breeding season (Figure 1). The ARUs were Song Meter SM4 (*n* = 1024 hexagons) or Song Meter Mini (*n* = 3 hexagons; Wildlife Acoustics, Maynard, MA) which are portable, weatherproof, and easily programmable. The SM4s had two built‐in omni‐directional microphones with signal‐to‐noise ratio of 80.0 dB typical at 1 kHz, two SDHC/SDXC flash card slots, an average of 543 h of recording battery life, and a recording bandwidth of 0.02–48.00 kHz at levels of −33.5 to 122.0 dB. The Song Meter Mini recorded at the same bandwidth, signal‐to‐noise ratio of 78.0 dB, one omni‐directional microphone, one SDHC/SDXC flash card slot, and 210–1040 h of battery life depending on configuration. We programmed all ARUs to record at a sampling rate of 32 kHz and saved files in single‐channel (mono) 16‐bit signed integer WAV format. These ARU models recorded owl calls with equivalent sensitivity to normal range of human hearing, and their effective recording radius is typically limited to approximately 250 m (19.6 ha) due to sound attenuation influenced by external factors such as terrain, vegetation, wind, and rain (Hane et al., 2022).

We mounted ARUs 1.5–2.0 m high on small trees (15–20 cm dbh) to allow microphones to extend past the bole for unobstructed recording. Most ARUs were deployed following a flexible standardized layout designed to maximize independence and representativeness: one station near the hexagon center and three positioned in non‐adjacent triangular locations within the interior (Figure 1). All stations were spaced ≥500 m apart, and located ≥200 m from the hexagon edge, to maximize coverage of the hexagon. Stations were placed on mid‐to‐upper slope positions to optimize sound transmission and positioned ≥50 m from roads, trails, and streams to reduce vandalism, theft, and excessive background noise. We programmed ARUs to record for approximately 10 h per day: 1‐h before sunset to 3‐h after sunset (4‐h sunset block), 2‐h before sunrise to 2‐h after sunrise (4‐h sunrise block), and for the first 10 min of every hour outside the sunrise and sunset blocks. This schedule concentrated effort during periods when spotted owls are most vocally active while subsampling coverage throughout the diel cycle (Duchac et al., 2020). The deployment of ARUs and data retrieval were the first two steps in our general workflow for data collection and processing (Lesmeister & Jenkins, 2022; Ruff et al., 2021).

### Acoustic data processing

We processed passive acoustic recordings using PNW‐Cnet v5, a convolutional neural network developed for automated identification of wildlife vocalizations in the Pacific Northwest (Lesmeister et al., 2026). The model classifies spectrograms (visual representations of sound) generated from 12‐s audio segments (hereafter, clips) and was trained on over 824,000 labeled examples representing 135 sound classes, including northern spotted owl and barred owl call types, other forest and domestic species, and anthropogenic sounds. PNW‐Cnet was implemented in Python using the Keras application programming interface (Watson et al., 2024) to Google's TensorFlow library (Abadi et al., 2015) and integrated into a workflow for large‐scale passive acoustic monitoring (Lesmeister et al., 2026; Ruff et al., 2021).

Model performance was evaluated using held‐out test sets and field‐based validation, achieving high precision for most sound classes at thresholds between 0.50 and 0.95 (Lesmeister et al., 2026). For northern spotted owl four‐note territorial calls, precision and recall at a 0.50 confidence threshold were 0.87 and 0.80, respectively; at 0.95, precision increased to 0.98 but recall declined to 0.36. To minimize false negatives, we adopted a 0.50 threshold and manually validated all positive detections. We also reviewed *Strix* spp. “bark” calls and spotted owl series call sound classes at the 0.95 confidence threshold (precision: 0.99 and 0.91 and recall: 0.37 and 0.50, respectively) in each survey week at all ARUs for pair context.

Barred owl detection was handled similarly to northern spotted owls. PNW‐Cnet v5 includes multiple barred owl vocalization classes (e.g., eight‐note, inspection, and series calls), all of which exhibited high performance in validation tests. For the eight‐note call, the most stereotyped and frequent vocalization, precision was 0.84 and recall was 0.83 at 0.50 threshold, and precision increased to 0.98 at 0.95 threshold with recall declining to 0.48 (Lesmeister et al., 2026). We confirmed the presence of eight‐note calls for each survey week at all ARUs stations to ensure no false positives were present in barred owl encounter histories and subsequently used the number of apparent detections at the 0.95 threshold to develop a calling intensity index for sites with confirmed detections. Based on PNW‐Cnet's relatively high recall for barred owl call, and the high vocal activity exhibited by barred owls, we judged that detectability was sufficiently high that naïve occupancy could be used as a covariate in northern spotted owl occupancy models without introducing substantial bias from false negatives.

Final weekly detection/non‐detection encounter histories for northern spotted owls included only confirmed vocalizations, ensuring high data integrity for occupancy modeling. This approach enabled efficient processing of millions of acoustic clips while maintaining rigorous standards for accuracy in estimating northern spotted owl landscape use and pair occupancy.

Clips containing confirmed northern spotted owl four‐note territorial calls underwent further analysis to predict the sex of vocalizing owls based on call frequency and duration metrics using a linear predictive model (Dale et al., 2022). Calls with the entire 95% prediction interval (PI) <0.5 were classified as female, calls with a PI >0.5 as male, and ambiguous calls (PI overlapping 0.5) as unknown sex. Females are less likely to use four‐note calls and vocalize with the bark series more often (Forsman et al., 1984). Therefore, for hexagons containing over 100 confirmed northern spotted owl calls, or any exhibiting evidence of female or paired vocalizations, we used additional expert review to search for female calling (e.g., barks or duets) in addition to four‐note calls.

### Censoring call‐back survey data

Call‐back surveys for northern spotted owls were frequently conducted within our study area by technicians involved in other research or conducting clearance surveys for land management projects. These surveys involved broadcasting recorded vocalizations of northern spotted owls for 10 min at call stations at 2‐min intervals to elicit territorial responses. We used three methods to identify and filter out detections resulting from these surveys. First, we provided surveyors with a brief series of pure‐tone sounds (1‐s each at frequencies of 0.5, 1.5, and 1.0 kHz) to play at a consistent volume immediately before or after their call‐back broadcasts. Lesmeister et al. (2026) trained the PNW‐Cnet v5 model to detect these survey tones with high performance (recall = 0.97, precision = 1.0). We manually validated all clips flagged by PNW‐Cnet v5 with prediction scores ≥0.95 for the presence of the survey tone. Second, we acquired data on locations and times of call‐back surveys conducted within or near our sampling hexagons. Third, surveys were typically distinguishable by being a consistent call type at approximately 2‐min intervals or continuous loop. We excluded detections of northern spotted owls in a hexagon from our analyses if a call‐back survey was confirmed or suspected to have occurred in that hexagon within the same 24‐h period, or if auditory evidence clearly indicated a broadcast call‐back recording.

After censoring data associated with call‐back survey data, we binned data into 1‐week survey occasions for the survey period and calculated the latency to detection, which was the number of days from the start of a passive acoustic survey to the first confirmed detection of a northern spotted owl four‐note territorial call. We also calculated the average number of northern spotted owl detections per hour of audio. Together, we used these measures to quantify differences in northern spotted owl vocal activity among hexagons and regions.

### Covariates

We selected hexagon and survey covariates (Table 1) based on previous research into northern spotted owl occupancy and habitat relationships (Appel et al., 2023; Davis et al., 2011, 2022; Duchac et al., 2020; Forsman et al., 1984; Weldy et al., 2023). Based on these hypotheses, we assigned the covariates to be used on one or more model parameter structures to model the occupancy and detection processes (Table 1). We standardized covariates and tested covariate correlations (Appendix S1: Tables S1–S3) to ensure no correlated covariates (>0.6) were included in the same parameter structure of a model due to concerns about multicollinearity (MacKenzie et al., 2018). We anticipated regional differences in landscape use, pair occupancy, and detection probabilities due to documented variation in northern spotted owl population trajectories, with lower landscape use and pair occupancy rates expected in northern regions and areas with high barred owl densities (Franklin et al., 2021; Wiens et al., 2021; Yackulic et al., 2019). To account for regional variation, we calculated the latitude of each hexagon centroid (LAT) and used a categorial variable for modeling region (MR), derived from a modified classification based on ecological similarities of physiographic provinces (Davis et al., 2022).

**TABLE 1:** Description of 5‐km^2^ hexagon and survey covariates used in multistate occupancy modeling of northern spotted owls.

Variable	Description	Model parameter
STATE	Observed state: state 0 (non‐detection, no four‐note calls); state 1 (single owl detected); state 2 (pair detected)	*p*
ARU	Type of autonomous recording unit used to conduct the survey, SM4 or Song Meter Mini	*p*
DATE	Ordinal date of day one of survey week	*p*, δ
ln(effort)	Natural log of hours of recording per week, calculated as total from all stations within hexagon	*p*, δ
PPRCP	Total weekly precipitation at hexagon (in millimeters) (PRISM Climate Group, 2024)	*p*, δ
PPRCPmn	Mean daily precipitation within hexagon per week (in millimeters) (PRISM Climate Group, 2024)	*p*, δ
SPL	Weekly mean of mean daily background noise as sound pressure level (dBFS)	*p*, δ
TEMPmn	Mean daily temperature (in degrees Celsius) (PRISM Climate Group, 2024)	*p*, δ
TEMPmx	Weekly mean max daily temperature (in degrees Celsius) (PRISM Climate Group, 2024)	*p*, δ
BO	Detection/non‐detection of barred owl eight‐note territorial calls binary for each hexagon and for weekly survey occasions	*p*, δ, ψ, *R*
BO‐PC1	Barred owl vocalization intensity index from a principal components analysis of the mean weekly and season long barred owl calling intensity, and number of stations and number of monitoring days with barred owl detections	*p*, δ, ψ, *R*
BOtime	Time since nearest demographic study area reached >1/3 occupancy of barred owls clustered by modeling region	ψ, *R*
ELEVsd	SD of elevation within hexagon (in meters) as a measure of topographic ruggedness (30 m resolution)	*p*, δ, ψ, *R*
ELEVmn	Mean elevation (in meters; 30 m resolution)	ψ, *R*
TPI270	Mean hexagon value of multiscale topographic position index at 270 m (Theobald et al., 2015)	*p*, δ, ψ, *R*
TOPODIV	Mean hexagon value of topographic diversity, scaled by 10; index derived from multi‐scale topographic position index and continuous heat‐insolation load index (Schloss et al., 2022; Theobald et al., 2015)	*p*, δ, ψ, *R*
MR	Modeling region: regional grouping classification of six areas based on a modification based on ecological similarities of the physiographic provinces (Figure 1; Davis et al., 2022).	*p*, δ, ψ, *R*
LAT	Latitude of hexagon centroid (decimal degrees [DD] × 1000)	δ, ψ, *R*
FOREST‐PC1	Principal component 1 from a principal components analysis of mean canopy cover of conifer, density of conifers greater than 50‐cm dbh, density of conifers greater than 75‐cm dbh, basal area weighted mean diameter of live conifers.	ψ, *R*
NR_INT	Proportion of hexagon with interior nesting and roosting forest (30 m resolution; Davis et al., 2022)	ψ, *R*
NRp	Proportion of hexagon covered with nesting and roosting forest (30 m resolution; Davis et al., 2022)	ψ, *R*
OGSImn	Mean old‐growth structure index (0–100) within hexagon. Calculated from abundance of large live trees, snags and down wood, and diversity of tree sizes (30 m resolution; https://lemma.forestry.oregonstate.edu/data/structure‐maps).	ψ, *R*
OGSI80	Proportion of hexagon classified as late‐successional and old‐growth forests with characteristics of stands >80 years old (30 m resolution)	ψ, *R*
CC_HDW	Mean canopy cover of all hardwoods (in percentage), including mid‐stories in mixed conifer‐hardwood forests, calculated using methods in the forest vegetation simulator (Crookston & Stage, 1999) (30 m resolution)	ψ, *R*
FRmn	Mean fire refugia index value within hexagon (Krawchuk et al., 2023)	ψ, *R*
FRp	Proportion of hexagon with fire refugia index (90 m resolution) value above 0.31	ψ, *R*
CHILI	Mean continuous heat‐insolation load index, scaled by 10 (Theobald et al., 2015)	ψ, *R*
CLIMWD	Mean climatic water deficit index, scaled by 10 (Reilly et al., 2024)	ψ, *R*

*Note*: Variables include both environmental predictors of model parameters: landscape use probability (ψ), probability that northern spotted owls were paired if present (*R*), probability of detecting any northern spotted owl (*p*) and probability of detecting evidence of a pair, if present (δ). Covariates were derived from topographic, climatic, and vegetation data sources and reflect ecological and acoustic conditions. Continuous covariates were standardized on unit scale by subtracting the mean of the variable and dividing the result by the variable's SD (Appendix S1: Table S1).

#### Hexagon covariates

Our hexagon covariates (Table 1) included old forest characteristics associated with northern spotted owls roosting and nesting behaviors, based on vegetation covariates derived from gradient nearest‐neighbor datasets (Bell et al., 2023). We summarized the mean old‐growth structural index (OGSImn), the proportion of each hexagon classified as late‐successional and old‐growth forests >80 years old (OGSI80), and mean canopy cover of hardwoods (CC_HDW). Using a principal components analysis we calculated an additional integrated index of mature coniferous forest conditions as the first principal component (FOREST‐PC1; Table 1) combining mean conifer canopy cover, densities of conifers greater than 50 cm and 75 cm dbh, and basal area weighted mean diameter of live conifers (PC1 SD = 1.77, proportion of variance explained = 0.78).

Because northern spotted owls rely heavily on old, structurally complex forests with large trees for nesting and roosting (Davis et al., 2022; Sovern et al., 2019; Wilk et al., 2018), we summarized the proportion (NRp) and area of interior (NR_INT) nesting‐roosting forest within each hexagon (Table 1). We also tested the ecological importance of fire refugia, areas used by northern spotted owls and less susceptible to high‐severity wildfire (Lesmeister et al., 2019; Lesmeister, Davis, et al., 2021). For fire refugia, we calculated the mean fire refugia index value (FRmn) and the proportion (FRp) of each hexagon categorized as fire refugia (values >0.31; Krawchuk et al., 2023).

For topographic covariates (Table 1) we calculated mean elevation (ELEVmn) and elevation SD (ELEVsd; i.e., ruggedness) for each hexagon. We also calculated mean multiscale topographic position index (TPI270), continuous heat‐insolation load index (CHILI, 270 m resolution), and topographic diversity (TOPODIV), an index derived from both TPI270 and CHILI (Schloss et al., 2022; Theobald et al., 2015). We included climatic water deficit (CLIMWD), which is an index reflecting a gradient from cool‐wet to hot‐dry conditions (Reilly et al., 2024).

To quantify the effect of barred owls on landscape use, occupancy, and detection probability, we incorporated multiple covariates describing barred owl presence and calling intensity (Table 1). For these covariates, we used barred owl detection counts predicted by PNW‐Cnet v5 (precision > 0.98), classified at a 0.95 confidence score threshold within surveys where ≥1 detection was human validated. These included human validated detection/non‐detection of barred owl eight‐note territorial calls binary for each hexagon and for weekly survey occasions (BO). Additionally, we generated an integrated barred owl calling intensity index using the first principal component (BO‐PC1; Table 1) from a principal components analysis on four highly correlated barred owl vocalization metrics: mean weekly barred owl calling intensity (eight‐note territorial calls per hour of recording), number of stations with barred owl presence, total station‐days with barred owl detections, and number of days with barred owl calling (PC1 SD = 1.74, proportion of variance explained = 0.76). Lastly, we included a covariate for time since barred owl invasion (BOtime) by modeling region, measured as mean years elapsed since one‐third of northern spotted owl territories in demographic study areas were occupied by barred owls (Yackulic et al., 2019).

#### Survey covariates

We developed survey covariates to quantify factors potentially influencing northern spotted owl detection probabilities (Table 1). Passive acoustic monitoring often encounters variation in detection probabilities related to background noise for many species, including northern spotted owls (Duchac et al., 2020). Therefore, we used Kaleidoscope Pro software (Wildlife Acoustics, Maynard, MA) to quantify weekly background noise levels by calculating sound pressure levels (SPL) within the 250–1000 Hz frequency bands where northern spotted owl territorial calls occur (Table 1). We calculated daily average SPL at each station, aggregated these to weekly station means, and then averaged across stations within hexagons for weekly hexagon‐level noise metrics. We conducted two tests for an ARU type effect on detection probabilities, an additive effect and a modeling region specific ARU effect because Song Meter Minis were only used in the Oregon Coast Range.

Because sampling effort significantly impacts acoustic survey detectability, we summarized total hours recorded per hexagon per week, log‐transforming the data to capture nonlinear relationships [ln(effort)], expecting detection probabilities to plateau after reaching an effort threshold (Table 1). To test seasonal differences in detection probability, we included a covariate for ordinal date of survey week (DATE). Weekly weather covariates included total weekly precipitation (PPRCP), mean daily precipitation (PPRCPmn), and daily mean (TEMPmn) and maximum (TEMPmx) temperatures within hexagons (PRISM Climate Group, 2024). We also examined the impact of terrain ruggedness on sound propagation by including elevation SD and topographic diversity as covariates influencing detection probabilities.

### Modeling strategy

We employed a multistate occupancy modeling approach using RPresence (MacKenzie et al., 2018; MacKenzie & Hines, 2023) to analyze passive acoustic monitoring data collected in 2023, focusing exclusively on northern spotted owl landscape use and pair occupancy. We conducted analyses at the hexagon scale, with weekly detection histories pooled across all ARU survey stations within each hexagon, accounting for staggered survey initiation (starting ordinal date 58). We modeled occupancy using three states:

*State 0*: vacant (no use by northern spotted owls)

*State 1*: single use (≥1 northern spotted owl uses the hexagon but no pair occupancy)

*State 2*: pair occupancy (the hexagon is assumed to overlap a territory of a mated pair of northern spotted owls)

States are assigned or partially assigned based on three types of observed events, listed in increasing order of certainty: no confirmed spotted owl four‐note calls (which maps to states 0, 1, or 2), ≥1 male or unknown sex four‐note call detected, or female four‐note call detected without any male detections (which map to states 1 or 2), and ≥1 female four‐note or bark call detected concurrently with ≥1 male four‐note call (which maps to state 2 only).

We used the conditional probability parameterization described by Nichols et al. (2007) to estimate the probability of landscape use by northern spotted owls regardless of pair state (ψ) and the probability that northern spotted owls were paired (female and male use), given that they were using a hexagon (*R*). Therefore, we did not assume that a hexagon used by a single northern spotted owl represented a territory, but if used by a pair we assumed the hexagon overlaps with a territory. Our models allowed for different state‐specific detection probabilities (*p*) and included a classification parameter (δ) to account for the probability of failing to detect both sexes, usually the female, when the true state is a pair present (i.e., 1 − δ). Therefore, δ can be interpreted as the approximate probability of successfully detecting a female at pair‐occupied sites. We predicted that the probability of detecting at least one northern spotted owl would be higher for hexagons in state 2, but those detections would primarily be males due to calling behavior differences by sex (Forsman et al., 1984). We fit models containing real parameters to the data with associated hypothesized model coefficients (β parameters), and defined model parameters as:

ψ_*i*_: probability that hexagon *i* is used by at least one northern spotted owl.

*R*_*i*_: probability that hexagon *i*, used by at least one northern spotted owl, is used by a pair (conditional pair use).

δ_*i*,*j*_: probability that evidence of northern spotted owl pair occupancy is detected in survey occasion *j* at hexagon *i*, given that is used by a pair.

*p*1_*i*,*j*_: probability that territorial vocalizations are detected in survey occasion *j* for a hexagon *i* used by a single (unpaired) northern spotted owl (i.e., given the hexagon is in state 1).

*p*2_*i*,*j*_: probability that territorial vocalizations are detected in survey occasion *j* for a hexagon *i* used by both male and female (paired) northern spotted owl (i.e., given the hexagon is in state 2).

### Model selection

Model selection followed the secondary candidate set strategy outlined by Morin et al. (2020), where subsets of models (*p*, δ, ψ, *R*) were developed independently in an information theoretic framework and most‐supported models from each subset were subsequently combined for a final stage of model selection. For sub‐model selection, we modeled non‐focal parameter structures as a function of modeling region and for detection probability (*p*) we also included state (single, pair) and effort [ln(effort)] covariates in the non‐focal structure.

We developed and performed a modified goodness‐of‐fit test (MacKenzie & Bailey, 2004) using a Pearson χ^2^ statistic with 100 bootstraps for our most‐supported multistate model to assess unmodeled heterogeneity across surveys (Appendix S1). We found evidence of overdispersion $\left(\hat{c}=1.26\right)$, so we adjusted information theoretic rankings to quasi‐Akaike information criterion (QAIC) and adjusted all SE based on the estimated variance inflation factor, $\hat{c}$ (Burnham & Anderson, 2002). We used QAIC values to assess model support and modeled covariates with a logit link function.

#### Model selection stages

We first modeled detection probability (*p*), evaluating covariates such as modeling region, ordinal date, weather conditions, barred owl presence and call intensity, background noise, sampling effort, and topography. We modeled state‐specific detection probabilities (*p*1 and *p*2) with an additive intercept because previous analyses confirmed distinct detectability differences between these states (Appel et al., 2023). We included effort [ln(effort)] in all models to account for any differences due to equipment failure. We tested the effect of covariates influencing correct classification of pairs (δ; i.e., the probability of detecting and correctly classifying both members of a pair when present), including precipitation, barred owl calling intensity, and date.

We modeled the probability of landscape use (ψ) and the probability that a used hexagon was in state 2 (pair occupancy; *R*), initially using univariate models and subsequently refining models with combinations of supported covariates based on QAIC selection. Due to the range and diversity of conditions present in our dataset, we tested additive and interactive effects of modeling region or latitude on landscape use parameters. We used no more than one interaction term per model for ease of interpretability and model stability. Final landscape use and conditional pair occupancy sub‐models included combinations of latitude, regional barred owl metrics, topographic variables, and forest structure indices.

At each stage of model selection, we retained parameter structures with ΔQAIC <10 for each sub‐model with covariates that added information (Arnold, 2010) and included these structures in our final candidate model set (Tables 2 and 3). We conducted all analyses in R (R Core Team, 2020) and report covariate beta estimates (β), odds ratios, SE, and 95% CI.

**TABLE 2:** Most supported sub‐models (>99% cumulative model weight) ranked by quasi Akaike information criterion (QAIC) value, model weight (*w*), and twice the negative log‐likelihood value (−2l).

Model	−2l	QAIC	*K*	∆QAIC	*w*
*p*					
*p*(STATE + ln(effort) + MR)	4245.06	3419.10	25	0.00	1.00
δ					
δ(MR + TPI270)	4232.57	3411.18	26	0.00	0.76
δ(MR + PPRCP)	4237.48	3415.08	26	3.90	0.11
δ(MR + BO)	4241.83	3418.53	26	7.35	0.02
δ(MR)	4245.06	3419.10	25	7.92	0.01
ψ					
ψ(LAT + TOPODIV + OGSImn)	4184.55	3367.07	23	0.00	0.97
ψ(BOtime + TOPODIV + OGSImn)	4194.61	3375.06	23	7.99	0.02
*R*					
*R*(LAT + ELEVsd + OGSImn)	4202.93	3383.66	24	0.00	0.51
*R*(LAT + CC_HDW + LAT × CC_HDW + OGSImn)	4200.66	3383.86	25	0.20	0.46
*R*(BOtime + ELEVsd + OGSImn)	4211.51	3390.47	24	6.81	0.02
*R*(LAT + CC_HDW + LAT × CC_HDW + ELEVsd)	4210.75	3391.87	25	8.21	0.01

*Note*: All combinations of these parameter structures were carried forward and combined in the final candidate model set. We fit models to evaluate covariates affecting probability of detecting any northern spotted owl (*p*), probability of detecting evidence of a pair, if present, during a survey period (δ), landscape use probability (ψ), and probability of pairing given hexagon is used (*R*) of northern spotted owls throughout their range in 2023. Refer to Table 1 for covariate descriptions and Appendix S1: Table S5 for full sub‐model candidate sets.

Abbreviations: *K*, number of model parameters; ∆QAIC, distance from lowest QAIC in sub‐model set.

**TABLE 3:** The 90% confidence set (i.e., cumulative model weight) of the final model selection stage, ranked by quasi Akaike information criterion (QAIC) value, model weight (*w*), and twice the negative log‐likelihood value (−2l).

Model	−2l	QAIC	*K*	∆QAIC	*w*
ψ(LAT + TOPODIV + OGSImn) *R*(LAT + CC_HDW + LAT × CC_HDW + OGSImn) *p* ^a^ δ^b^	4144.52	3337.30	24	0.00	0.48
ψ(LAT + TOPODIV + OGSImn) *R*(LAT + ELEVsd + OGSImn) *p* ^a^ δ^b^	4149.89	3339.56	23	2.26	0.16
ψ(LAT + TOPODIV + OGSImn) *R*(LAT + CC_HDW + LAT × CC_HDW + ELEVsd) *p* ^a^ δ^b^	4147.75	3339.87	24	2.56	0.13
ψ(LAT + TOPODIV + OGSImn) *R*(BOtime + ELEVsd + OGSImn) *p* ^a^ δ^b^	4151.98	3341.23	23	3.92	0.07
ψ(LAT + TOPODIV + OGSImn) *R*(LAT + CC_HDW + LAT × CC_HDW + OGSImn) *p* ^a^ δ^c^	4150.38	3341.95	24	4.64	0.05
ψ(LAT + TOPODIV + OGSImn) *R*(LAT + CC_HDW + LAT × CC_HDW + ELEVsd) *p* ^a^ δ^c^	4152.81	3343.88	24	6.58	0.02

*Note*: We fit models to evaluate covariates affecting probability of detecting any northern spotted owl (*p*), probability of detecting evidence of a pair, if present, during a survey period (δ), landscape use probability (ψ), and probability of pairing given hexagon is used (*R*) of northern spotted owls throughout their range in 2023. Refer to Table 1 for covariate descriptions and Appendix S1: Table S6 for full model set.

Abbreviations: *K*, number of model parameters; ∆QAIC, distance from lowest QAIC in final model set.

^a^ *p*(STATE + ln(effort) + MR).

^b^ δ(MR + TPI270).

^c^ δ(MR + PPRCP).

### Derived parameters

We generated derived parameters from the most‐supported final model and estimated SE for those parameters using the delta method (Powell, 2007), to explore variation in pair occupancy and detection probability across our sampled hexagons. We computed the following derived parameters (MacKenzie et al., 2018):

$${\text{ϕ0}}_{i}=1-\psi_{i}:\text{probability of hexagon}\;i\;\text{being in state}\;0=\mathrm{Pr}\left(\text{absence}\right),$$

$${\text{ϕ1}}_{i}=\psi_{i}\times \left(1-R_{i}\right):\text{probability of hexagon}\;i\;\text{being in state}\;1=\mathrm{Pr}\left(\text{single}\;\mathrm{use}\right),$$

$${\text{ϕ2}}_{i}=\psi_{i}\times R_{i}:\text{probability of hexagon}\;i\;\text{being in state}\;2=\mathrm{Pr}\left(\text{pair occupancy}\right),$$

$$p{\left[\text{pair}\right]}_{i,j}:p2_{i,j}\times \delta_{i,j}=\mathrm{Pr}(\text{detecting}\;a\;\text{pair}\;\mathrm{at}\;\text{hexagon}\;i\;\text{in survey occasion}\;j\;\text{given}\;a\;\text{pair is present}).$$

Using Bayes' theorem, we estimated, within a survey across *n* surveys, the probability of only detecting a single sex in a hexagon that was occupied by a pair, $\varepsilon =\mathrm{Pr}\left(\text{pair}\;\mathrm{occ}\;|\;\text{only}\;a\;\text{single}\;\mathrm{sex}\;\text{detected}\geq 1\;\text{in}\;n\;\text{surveys}\right)$ as:

$$=\frac{\;\mathrm{Pr}\left(\text{only}\;a\;\text{single}\;\mathrm{sex}\;\text{detected}\geq 1|\;\text{pair}\;\mathrm{occ}\right)\times \mathrm{Pr}\left(\text{pair}\;\mathrm{occ}\right)}{\mathrm{Pr}\left(\text{only}\;a\;\text{single}\;\mathrm{sex}\;\text{detected}\geq 1\right)}$$

$$=\frac{\;\left(\left(1-{\left(1-p2\right)}^{n}\right)-\left(1-{\left(1-p2\times \delta \right)}^{n}\right)\right)\times \psi \times R}{\left(\left(1-{\left(1-p2\right)}^{n}\right)-\left(1-{\left(1-p2\times \delta \right)}^{n}\right)\right)\times \psi \times R+\left(1-{\left(1-p1\right)}^{n}\right)\times \psi \times \left(1-R\right)}$$

### Predictive maps

We used the most‐supported model from the final model set to generate predictive maps of northern spotted owl landscape use (ψ) and pair occupancy (ϕ2), for all forested hexagons (≥50% forest capable) across the study area (Appendix S1: Figure S1; MacKenzie et al., 2018). We created associated maps of modified CV (SE/mean), with SEs computed using the delta method (Powell, 2007), to map prediction uncertainty. We also report modeled and derived occupancy parameter (ψ, *R*, ϕ0, ϕ1, ϕ2) means and CV for each of the six modeling regions.

We interpreted pair occupancy probabilities (ϕ2) relative to a functional extinction threshold of 0.20, which has been widely applied in conservation contexts as an indicator of high demographic risk when abundance or vital‐rate data are unavailable (Laufenberg et al., 2018; Noon & McKelvey, 1996; Ovaskainen & Hanski, 2003). This threshold provides an operational metric for identifying regions where populations may no longer support viable breeding pairs.

To understand the degree to which northern spotted owl distribution in 2023 aligned with available nesting and roosting forest (Davis et al., 2022) in each forest capable hexagon, we subtracted estimates of ψ and ϕ2 from the proportion of the hexagon covered with nesting and roosting forest (NRp). These metrics are not equivalent; however, we consider this as a visual assessment of spatial mismatch between northern spotted owl landscape use and the presence of a recognized component of breeding habitat (Lesmeister et al., 2018). Strongly positive values represent areas used less than expected based on nesting forest alone, and areas with strongly negative values could represent areas where the model predicted higher use than expected based on the amount of nesting and roosting forest.

## RESULTS

We collected and processed a total of 2.12 million h of acoustic recordings from 4081 ARU stations distributed across 1027 hexagons in staggered 6–8‐week survey periods during February 24 through September 6, 2023 (Figure 1). Sampling covered approximately 3% of all forest‐capable hexagons and represented about 5% of available federally managed forest‐capable hexagons within the NWFP area (Appendix S1: Table S4). On average, ARUs in each hexagon recorded 269.80 h (SE = 1.79) per week, resulting in a total sampling effort ranging from 600 to 2368 h of audio per hexagon across the 8‐week survey period.

Following Ruff et al. (2021), we employed a semi‐automated detection pipeline and confirmed a total of 34,341 clips containing the northern spotted owl four‐note territorial call, 1208 clips containing the northern spotted owl bark call, and 9467 clips containing other northern spotted owl vocalizations. We confirmed northern spotted owl calls in 261 of the 1027 sampled hexagons (naïve landscape use = 25%). Of these 261 hexagons, 89 (34%) exhibited confirmed vocalizations from both male and female northern spotted owls, representing approximately 9% of all sampled hexagons (Appendix S1: Table S4). We observed a broad regional gradient in naïve landscape use ranging from 13% to 17% in Washington and Oregon Coast Range, 44% in the Oregon and California Klamath Mountains, to 91% (10 of 11 hexagons surveyed) for the California Coast Region (Appendix S1: Table S4). We detected no northern spotted owls in the 39 hexagons we surveyed north of latitude 47.7° N in the Washington Cascades (Figure 1).

The mean latency to first detection of northern spotted owl four‐note territorial calls was 13.20 days (SE = 0.86; range = 0–55 days). Latency varied considerably by region, with the shortest latency occurring in the California Coast region (mean = 0.70 days, SE = 0.60), approximately 12–36 times shorter than other regions. The Oregon and California Klamath Mountains region had the next shortest latency at 8.30 days (SE = 1.14), whereas the Oregon Coast Range region had the longest latency at 25.60 days (SE = 3.49). The mean latency measured in survey weeks across the full sampling range was 2.5 weeks (SE = 0.12).

The mean northern spotted owl calling intensity, measured as density of four‐note territorial calls per recording hour, was 0.02 calls/h (SE < 0.01). We observed considerable regional variation, with the highest calling intensity in the California Coast region (mean = 0.14 calls/h, SE < 0.01), while the lowest intensity recorded in the Oregon Coast Range region (mean = 0.002 calls/h, SE = 0.002).

Barred owls were far more prevalent than northern spotted owls, with presence confirmed at 891 hexagons (86.8% of sampled hexagons), and a total of 279,439 validated clips containing their eight‐note territorial call. Mean apparent weekly calling intensity for barred owls was 0.14 calls/h (SE < 0.01), approximately 8.1 times greater than northern spotted owl calling intensity. We also observed considerable regional variation in barred owl naïve landscape use rates with at least one barred owl detection in 100% of Oregon Coast Range hexagons, 94% of Washington Coast and Western Cascades hexagons, 45% of California Coast, and 72% of Oregon and California Klamath Mountains hexagons (Appendix S1: Table S4).

### Sub‐models

For state‐specific detection probability (*p*), the regional sub‐model was advanced to the final model set and was strongly supported (>27 QAIC improvement) compared to all other model structures without regional variation (Table 2; Appendix S1: Table S5). While not advanced for consideration in the final model set, univariate models for detection probabilities showed support for negative effects of increased background noise levels (β = −0.17, SE = 0.10) and precipitation (β = −0.38, SE = 0.10), and positive effect of temperature (β = 0.23, SE = 0.09; Appendix S1: Table S5).

**FIGURE 2:**
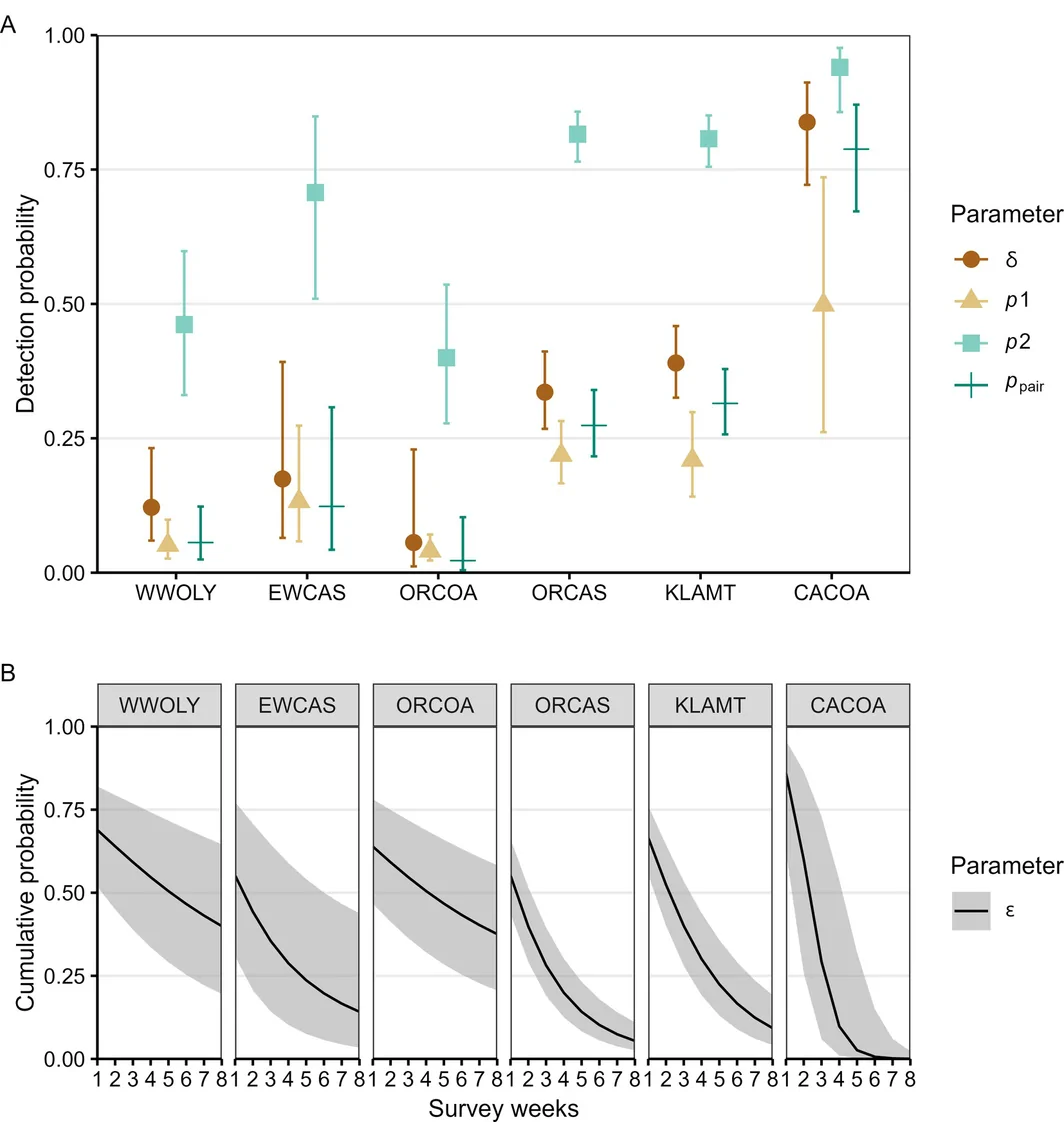
The estimated probability of detecting any northern spotted owl during a single hexagon‐week when the true state was paired (*p*2) was higher than when the true state was a single owl present (*p*1) for all modeling regions (panel A). The probability of detecting evidence of a pair, if present (δ) varied by modeling region. The associated probability of detecting both the male and the female of a pair (*p*_pair_ = *p*2 × δ) was highest in the California Coast (CACOA), moderate in the Oregon and California Cascades (ORCAS), and Oregon and California Klamath Mountains (KLAMT; panel A). The cumulative probability of only detecting the male in a hexagon when the true state was paired (ε) declined with weeks of surveys (panel B). Mean estimates and 95% CI were generated from the most‐supported model of multistate occupancy, SE were adjusted by overdispersion parameter, ĉ = 1.26, and generated with the delta method. EWCAS, Washington Eastern Cascades; ORCOA, Oregon Coast Range; WWOLY, Washington Coast and Western Cascades.

The conditional probability of detecting pair occupancy when present (δ) varied significantly by modeling region; this effect was included in all sub‐models carried forward to the final model set (Table 2; Figure 2). We found support for negative effects of multiscale topographic position index (β = −0.30, SE = 0.10) and total weekly precipitation (β = −0.47, SE = 0.20) on δ. There was also a positive association with barred owl weekly detections (β = 0.34, SE = 0.22; Table 2), which may be attributable to spotted owl pairs responding territorially to barred owl presence (Duchac et al., 2020; Wood et al., 2021). Models with ruggedness and date were included based on ΔQAIC ≤10. However, model coefficient CLs included zero; so, neither covariate was carried forward (Appendix S1: Table S5).

Covariates in the top two ψ sub‐models advanced to the final model set (Table 2). Northern spotted owl landscape use was positively associated with old‐growth forest structure index (β = 0.52, SE = 0.12) and topographic diversity (β = 0.67, SE = 0.13), negatively associated with increasing latitude (β = −0.78, SE = 0.14) and with time since barred owl invasion (BOtime; β = −0.80, SE = 0.16; Appendix S1: Table S5). The univariate model describing northern spotted owl nesting and roosting forest (NRp) indicated a similar, but less supported response (∆QAIC = 47.45, β = 0.39, SE = 0.11) compared to the correlated OGSImn (*r* = 0.66) that was moved forward and included in the final model set (Appendix S1: Table S5).

Covariates in the top four *R* sub‐models were advanced to the final candidate set (Table 2; Appendix S1: Table S5). The probability of pair occupancy given use decreased with latitude (β = −1.36, SE = 0.25) and time since barred owl invasion (β = −1.47, SE = 0.40). *R* was positively associated with ruggedness (ELEVsd; β = 0.91, SE = 0.23) and old growth forest structure index (β = 0.88, SE = 0.22).

### Final candidate set

The final candidate model set included 32 models (Appendix S1: Table S6). The most‐supported model [ψ(LAT + TOPODIV + OGSImn), *R*(LAT × CC_HDW + OGSImn), *p*(STATE + ln(effort) + MR), δ(MR + TPI270)] outperformed the second‐ranked model by 2.26 QAIC units and had a model weight of 0.48. The four most‐supported models (84% of the cumulative model weight) differed only in *R* sub‐model structure (Table 3).

#### Detection probabilities

Detection probabilities differed substantially by occupancy state. The odds of detecting any owl vocalizations when the true occupancy state was a pair (*p*2) was 15.81 (CI = 10.93–22.85) times higher than when only a single sex (typically male) used a hexagon (*p*1; Figure 2). Recording effort also increased weekly detection probability; specifically, an additional 40 h of recording effort (approximately one day of recordings at four stations) resulted in a 1.48‐fold increase in the odds of detection (CI = 1.47–1.64; Table 4).

**TABLE 4:** Coefficient estimates (β), SE, and 95% CI for covariates in the most‐supported final model (Table 3).

Parameter	Covariate	β	SE	95% CI	
ψ	Intercept	−1.12	0.17	−1.46	−0.78
ψ	TOPODIV	0.67	0.13	0.41	0.94
ψ	OGSImn	0.40	0.13	0.14	0.65
ψ	LAT	−0.69	0.14	−0.97	−0.41
*R*	Intercept	−1.91	0.36	−2.62	−1.21
*R*	LAT	−1.40	0.34	−2.07	−0.73
*R*	CC_HDW	−0.82	0.32	−1.45	−0.20
*R*	OGSImn	0.60	0.22	0.17	1.03
*R*	LAT × CC_HDW	−1.12	0.30	−1.71	−0.52
*p*	Intercept (*p*1 KLAMT)	−7.93	0.92	−9.74	−6.12
*p*	*p*2	2.76	0.19	2.39	3.13
*p*	ln(effort)	1.16	0.16	0.84	1.48
*p*	CACOA	1.32	0.51	0.32	2.33
*p*	EWCAS	−0.55	0.46	−1.45	0.34
*p*	ORCAS	0.05	0.21	−0.37	0.47
*p*	ORCOA	−1.84	0.32	−2.46	−1.21
*p*	WWOLY	−1.59	0.32	−2.22	−0.96
δ	INT(KLAMT)	−0.42	0.15	−0.71	−0.13
δ	CACOA	2.10	0.38	1.35	2.86
δ	EWCAS	−1.19	0.60	−2.35	−0.02
δ	ORCAS	−0.29	0.22	−0.71	0.14
δ	ORCOA	−2.35	0.84	−3.99	−0.71
δ	WWOLY	−1.75	0.45	−2.62	−0.87
δ	TPI270	−0.30	0.10	−0.49	−0.11

*Note*: Refer to Table 1 for covariate descriptions. Probability of detecting any northern spotted owl (*p*) if true state 1 (*p*1) or true state 2 (*p*2); probability of detecting evidence of a pair (δ); landscape use (ψ); probability of pairing given use (*R*).

Abbreviations: CACOA, California Coast; EWCAS, Washington Eastern Cascades; KLAMT, Oregon and California Klamath Mountains; ORCAS, Oregon and California Cascades; ORCOA, Oregon Coast Range; WWOLY, Washington Coast and Western Cascades.

Regional detection probabilities (*p*1, *p*2, and δ) varied significantly, with notably lower detectability in northern regions, particularly the Oregon Coast Range and Washington Coast and Western Cascades, and higher detectability in the southernmost regions such as Oregon and California Cascades, Oregon and California Klamath Mountains, and California Coast. The variations likely reflect a combination of regional differences in ecological conditions, invasive barred owl pressures, spotted owl home range size, spotted owl abundance, and potentially sound transmission (Table 4; Figure 2).

Throughout all regions, the cumulative probability of detecting only the male in hexagons that were occupied by a pair decreased with more sampling effort, but the rate of improvement varied substantially by region (Figure 2). Across all regions, fewer weeks of sampling resulted in higher probabilities of potentially mis‐assigning pair status based on observed survey results, meaning typically only the males were detected due to low female detection probability. The California Coast and Oregon and California Klamath Mountains showed the most rapid increases in detecting both members of the pair. In contrast, the two regions in Washington and the Oregon Coast Range exhibited slower change in cumulative probability of detecting both sexes (Figure 2), indicating persistent challenges in observing the true biological state in regions with small populations. By approximately 6 weeks of survey effort, the probability of detecting both sexes exceeded 0.8 in most regions but remained below 0.6 in the Washington Coast and Western Cascades and Oregon Coast Range regions (Figure 2).

#### Landscape use and pair occupancy

**FIGURE 3:**
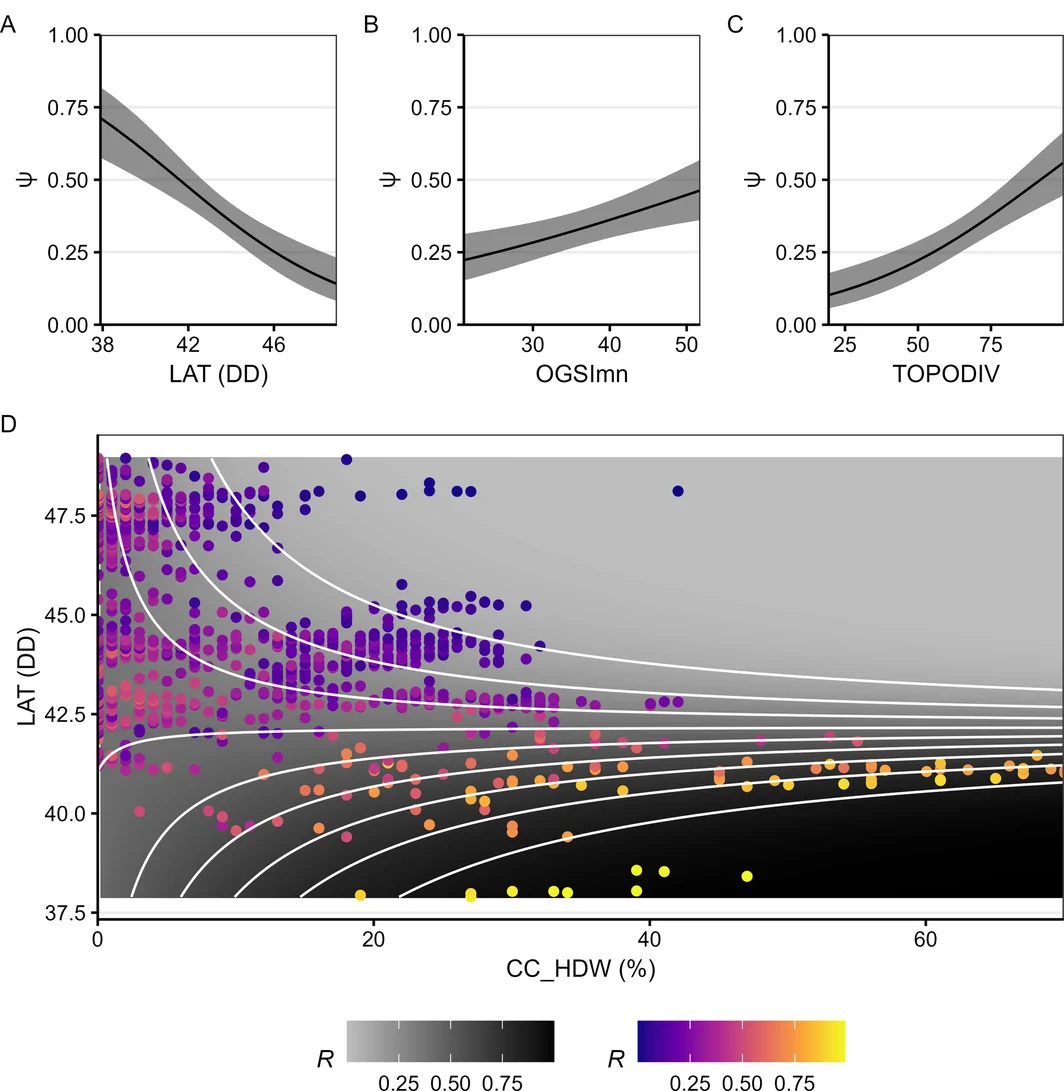
From the most‐supported model and holding other variables at their means, the predicted effect of latitude in decimal degrees (LAT; DD; panel A), mean old‐growth structural index (OGSImn; panel B), and topographic diversity (TOPODIV; panel C) on northern spotted owl landscape use (ψ). Panel (D) shows the direction and strength of effect for percent canopy cover of hardwoods (CC_HDW) on the conditional probability that a hexagon is used by a pair of northern spotted owls (*R*), which differs with latitude. The contour plot with black‐to‐white tile shows the predicted effect of the interactive effect of latitude and canopy cover of hardwoods on *R*. Point estimates (blue‐to‐yellow) represent the estimated mean parameter values from the actual sampled data, generated from the most‐supported multistate model of northern spotted owl occupancy in 2023. Refer to Table 1 for covariate descriptions.

Our most‐supported model indicated that northern spotted owl landscape use (ψ) and pair occupancy (ψ × *R*) were positively influenced by old‐growth structural complexity (OGSImn; β_ψ_ = 0.40, SE = 0.13; β_*R*_ = 0.60, SE =0.22) and negatively associated with latitude (β_ψ_ = −0.69, SE = 0.14; β_*R*_ = −1.40, SE = 0.34). Each SD (11 index units) increase of old‐growth structural complexity resulted in a 49% increase in the odds of landscape use (CI = 15%–91%; Figure 3), and an 82% increase in the odds of a used hexagon being occupied by a pair (CI = 19%–179%). A one SD (23 units) increase in topographic diversity was associated with a 96% increase in the odds of landscape use (CI = 50%–155%). With each two decimal degrees increase in latitude the odds of use decreased by 39% (CI = 25%–50%; Figure 3). The relationship between hardwood canopy cover and *R* varied latitudinally; hardwood canopy cover had a small negative effect in northern and mid‐latitudes, whereas in southern regions, increased hardwood canopy cover positively influenced pair occupancy (Figure 3; Table 4). The hardwood component in old forests increases substantially in the southern part of the northern spotted owl range but is distinct from pure hardwood forests due to prevalence of coniferous trees in these forests.

**FIGURE 4:**
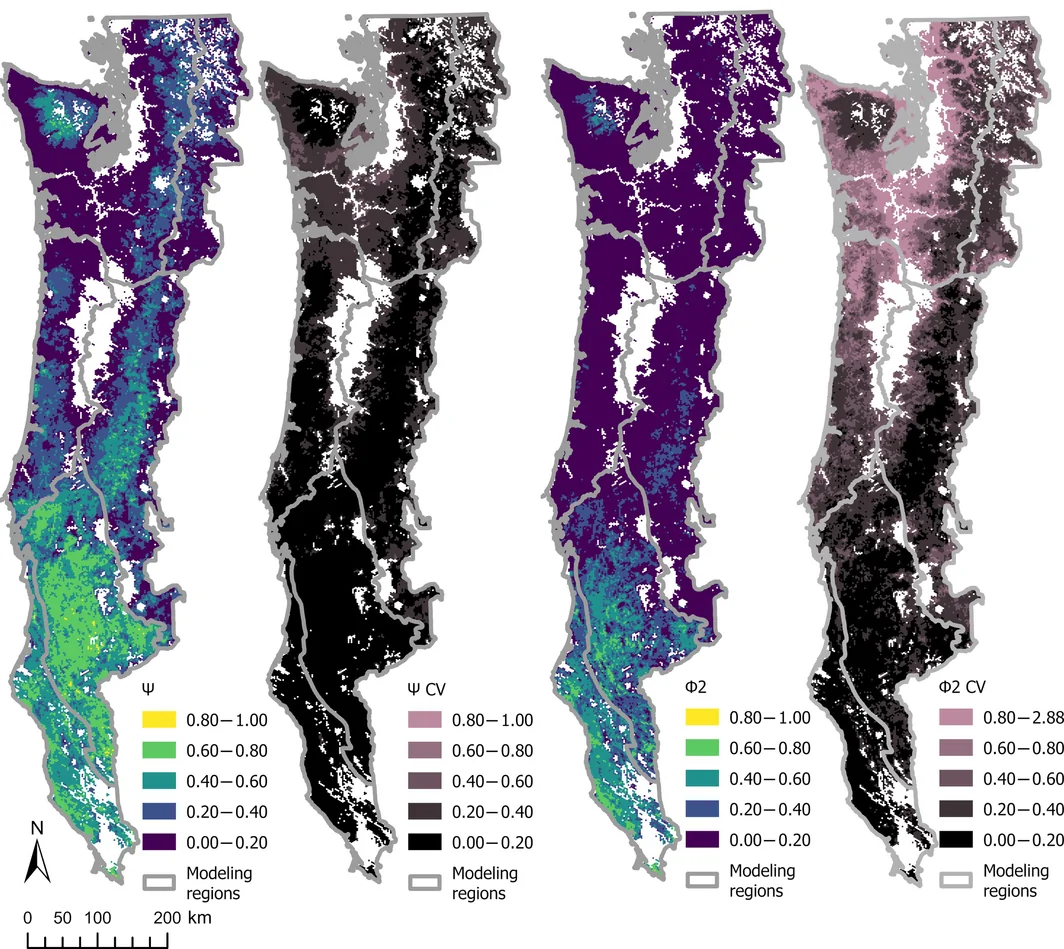
Mean and CV of predicted landscape use (ψ) and pair occupancy (ϕ2) across all forest capable hexagons from most‐supported multistate model of northern spotted owl occupancy. The SE used in CV calculations are adjusted by overdispersion parameter, *ĉ* = 1.26.

**FIGURE 5:**
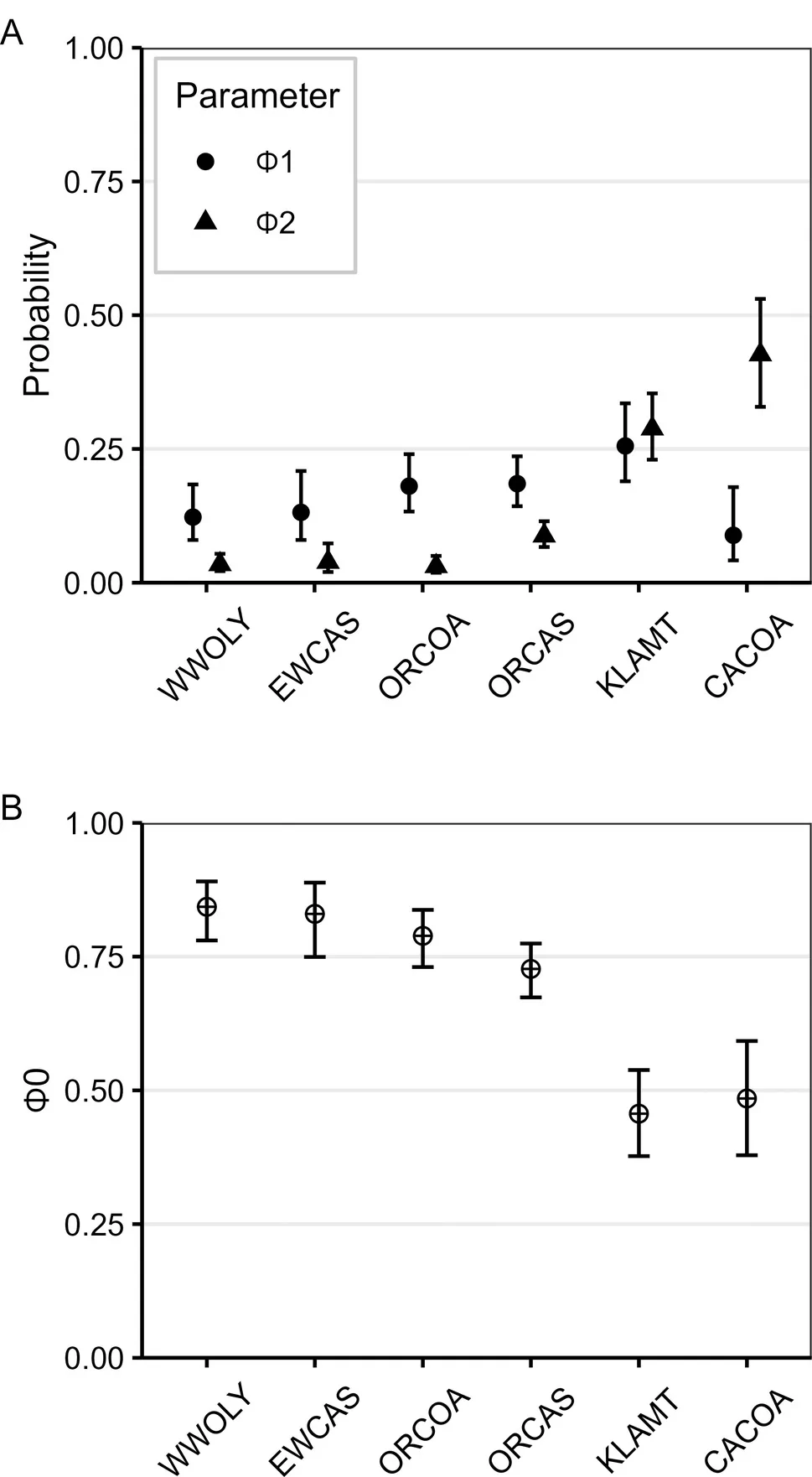
The mean and 95% CI for northern spotted owl landscape use by singles only (ϕ1; panel A), pair occupancy (ϕ2; panel A), and likelihood of absence (ϕ0 = 1 − ψ; panel B) projected across all modeling regions. Refer to Figure 1 for modeling region locations. CACOA, California Coast; EWCAS, Washington Eastern Cascades; KLAMT, Oregon and California Klamath Mountains; ORCAS, Oregon and California Cascades; ORCOA, Oregon Coast Range; WWOLY, Washington Coast and Western Cascades.

We observed pronounced regional differences in predicted landscape use and pair occupancy (Figure 4; Table 5). Regional estimates for pair occupancy fell well below the functional extinction threshold of 0.20 in four of our six modeling regions (Figure 5) and 67% of all forest capable hexagons had estimated pair occupancy 95% CLs below 0.2. Landscape use and pair occupancy estimates were highest in the California Coast but we had a small sample from this region (*n* = 11 hexagons) and seven of those hexagons were in Marin County where each hexagon was used and we detected only a few barred owl calls (Lesmeister et al., 2024). The next highest estimates were in the Oregon and California Klamath Mountains, regions characterized by the shortest barred owl residence time, complex landscapes with a mix of older mixed‐conifer and hardwood forests, and diverse topographic features favorable to northern spotted owl occupancy (Franklin et al., 2000). Conversely, northern regions, particularly the two regions in Washington and the Oregon Coast Range, displayed significantly lower predicted landscape use and pair occupancy (Table 5). The Olympic Peninsula of northwest Washington had higher landscape use and pair occupancy than other northern areas (Figure 4). The highest coefficients of variation for landscape use and pair occupancy were in regions with both low sampling density and low occupancy rates, particularly the low elevation forests of Oregon and Washington, implying higher relative variability and lower consistency in estimates (Figure 4). The out of sample estimates for nonfederal forested lands, prevalent in those low elevation regions, were consistently lower than sampled areas (Appendix S1: Figure S2). The mean probability of pair occupancy was higher than the probability of single (state 1) landscape use (i.e., ψ × [1 − *R*], such that *R* > 0.5) in the Oregon and California Klamath Mountains and California Coast regions but was lower in other regions of the study area where northern spotted owls are primarily absent (Figure 5).

**TABLE 5:** Mean (CV) model parameter estimates by modeling region for all forest capable 5‐km^2^ hexagons.

Modeling region	ψ	*R*	ϕ0	ϕ1	ϕ2
KLAMT	0.54 (0.07)	0.49 (0.09)	0.46 (0.08)	0.26 (0.13)	0.29 (0.10)
CACOA	0.52 (0.10)	0.81 (0.07)	0.48 (0.10)	0.09 (0.33)	0.43 (0.11)
EWCAS	0.17 (0.18)	0.21 (0.29)	0.83 (0.04)	0.13 (0.22)	0.04 (0.30)
ORCAS	0.27 (0.08)	0.28 (0.13)	0.73 (0.03)	0.19 (0.11)	0.09 (0.12)
ORCOA	0.21 (0.12)	0.11 (0.27)	0.79 (0.03)	0.18 (0.13)	0.03 (0.23)
WWOLY	0.16 (0.16)	0.11 (0.22)	0.84 (0.03)	0.12 (0.19)	0.03 (0.21)

*Note*: CV calculated as SE divided by mean, with SE generated with delta method.

Abbreviations: CACOA, California Coast; EWCAS, Washington Eastern Cascades; KLAMT, Oregon and California Klamath Mountains; ORCAS, Oregon and California Cascades; ORCOA, Oregon Coast Range; *R*, probability of pairing given hexagon is used (i.e., conditional pair use); WWOLY, Washington Coast and Western Cascades; ψ, probability of use by any northern spotted owl; ϕ0, probability of absence; ϕ1, probability of use by single northern spotted owl; ϕ2, probability of pair occupancy.

**FIGURE 6:**
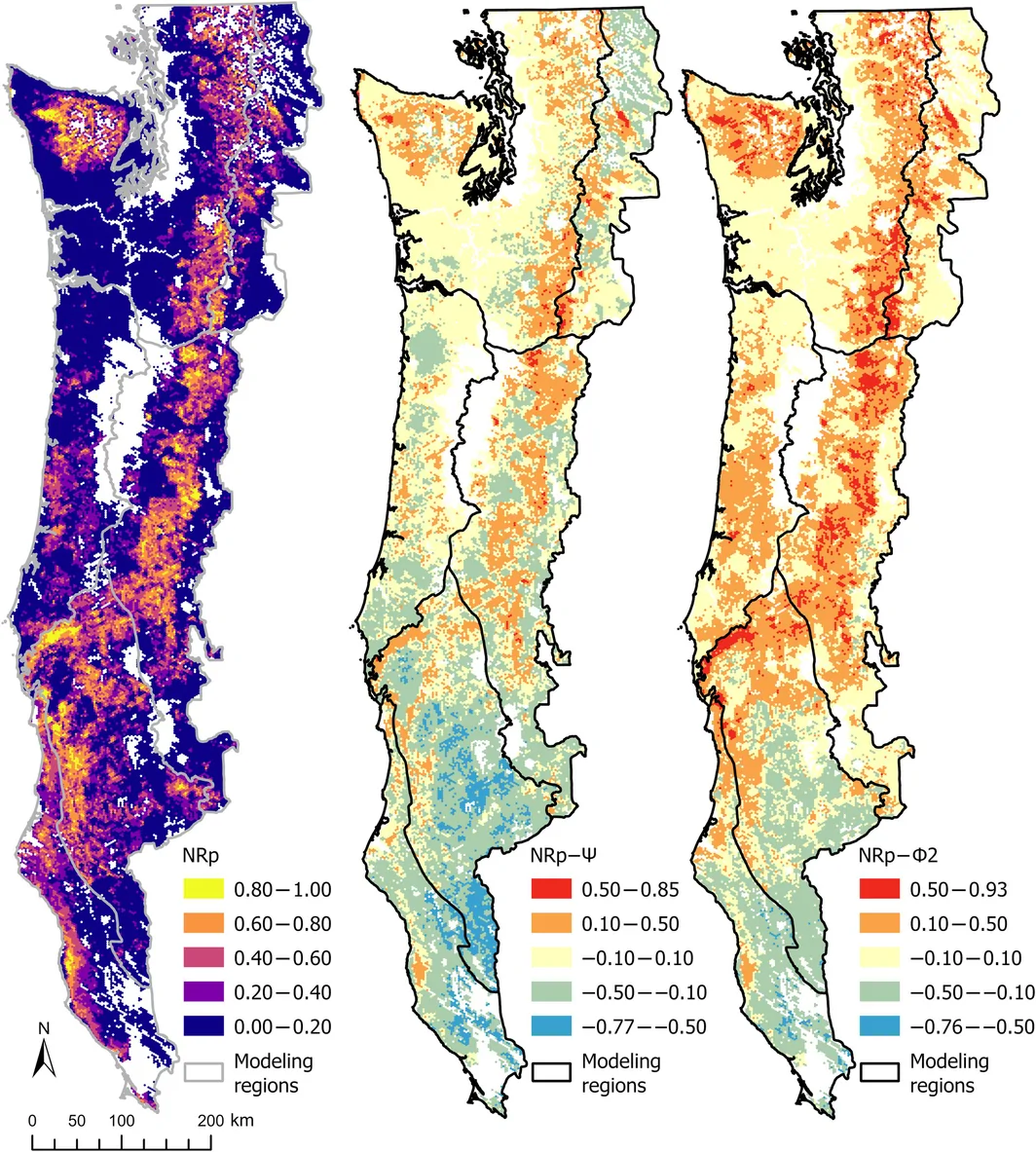
Proportion of northern spotted owl nesting and roosting forest (NRp) in forest capable 5‐km^2^ hexagons (left panel), and difference between NRp and the estimated landscape use (ψ; center panel) and pair occupancy (ϕ2; right panel). Strongly positive values (red) on center and right panels represent areas used less than expected based on the availability of nesting and roosting forest.

We observed regional patterns in the mismatch of northern spotted owl distribution and available nesting and roosting forest based on the difference between estimated landscape use and pair occupancy with the availability of nesting and roosting forest (NRp; Figure 6). Nesting and roosting forest was widespread throughout most of the federally managed forest lands, but despite its availability, northern spotted owl landscape use and pair occupancy were low in the Washington Cascades, Oregon Coast Range, and northern portion of the Klamath Mountains (Figure 6). The mismatch was especially high for pair occupancy.

## DISCUSSION

Range‐wide passive acoustic monitoring provides compelling evidence of extinction risks facing northern spotted owls due to invasive barred owls, complex habitat relationships, and significant methodological considerations regarding species detectability. Northern spotted owl landscape use and pair occupancy were lowest in regions in Washington followed by Oregon then California. Our observed regional variability in calling intensity (average number of calls per hour) and latency to first detection (average number of days from beginning of survey) provided additional metrics for assessing population status. The highest calling intensities occurred in Marin County where most of our sampling on the California Coast occurred (7 of 11 hexagons). These findings contrasted starkly with the lowest intensities in the Oregon Coast Range, but most of the California Coast is not under federal management and therefore not surveyed in this study (Figure 1). Low calling intensities and extended detection latencies in northern regions could indicate sparse populations nearing extinction thresholds. Notably, we detected no northern spotted owls north of latitude 47.7° N in the Washington Cascades and very few detections in the Oregon Coast Range. These areas are characterized by long‐established barred owl populations with high occupancy rates (Wiens et al., 2021; Yackulic et al., 2019).

### Extinction thresholds

These regional pair occupancy estimates for northern spotted owls suggest that populations in large portions of the range are at or near functional extinction thresholds (ϕ2 ≤ 0.20), a level widely recognized as indicative of high demographic risk (Noon & McKelvey, 1996; Ovaskainen & Hanski, 2003). Noon and McKelvey (1996) defined these thresholds based on the proportion of historically occupied territories, which were home‐range sized areas used by resident, territorial pairs as determined through mark‐capture‐recapture demographic studies. These territories did not include transient individuals or broader landscapes. Below this level, populations were considered at high risk of extinction because of demographic stochasticity and loss of connectivity leading to genetic isolation. In our study, the four northern regions had mean estimates of pair occupancy below 0.20. The Washington Cascades and the Oregon Coast Range consistently fell below this level, with average pair occupancy probability less than 0.05. Although our occupancy states differ conceptually because they represent 5‐km^2^ hexagons sampled for calling activity rather than demographic territories, they provide an analogous measure of pair presence (Weldy et al., 2023). These findings are consistent with extinction risk frameworks that emphasize the importance of severely reduced distribution and abundance when evaluating probable extinction or loss of functional populations (Butchart et al., 2006). Although individual northern spotted owls may persist, these regions likely no longer support viable breeding populations, requiring urgent management intervention to avoid regional extinction in the short term and entire subspecies in the long term.

The near‐total absence of northern spotted owls and high occupancy of barred owls in these regions underscores an imminent risk posed by barred owls (Jenkins et al., 2025; Wiens et al., 2021). Invasive species can drastically alter community dynamics, precipitating rapid biodiversity declines (Doherty et al., 2016). Barred owls have fundamentally altered competitive dynamics by aggressively displacing native northern spotted owls and competing directly for nest sites and prey (Jenkins et al., 2021; Jenkins, Lesmeister, Wiens, et al., 2019; Van Lanen et al., 2011; Wiens et al., 2014). Northern spotted owls are on the path to regional extirpation unless management interventions, such as barred owl removal, are implemented (Dumbacher & Franklin, 2024). However, creating suitably large reserves free of barred owls may not be feasible in regions like the Washington Cascades and Oregon Coast Range that have small northern spotted owl populations and high barred owl densities and occupancy rates (Franklin et al., 2021; Wiens et al., 2021). In these regions, an alternative strategy to prevent extinction, and preserve remaining genetic diversity, could be to bring the few remaining northern spotted owls into captivity for breeding populations (Hufbauer et al., 2015).

If demographic or genetic rescue is considered as a recovery option for spotted owls in the Washington Cascades and Oregon Coast Range, then evidence‐based tools can be used to evaluate the feasibility and effectiveness of these actions (IUCN/SSC, 2013). The process to establish successful captive breeding that produces individuals for reintroduction is likely to take decades and involve coordination across federal, state, tribal, and private landowners. For example, the Pacific fisher reintroduction program in the Washington Cascades did not include captive breeding but took over 20 years from inception to reintroductions (Lewis et al., 2022). The alarming patterns in occupancy rates we observed in this study and their relationship to a suggested extinction threshold (Noon & McKelvey, 1996) align with the conclusions of Yackulic et al. (2019) that the northern spotted owl was highly likely (>95% probability) to become extirpated (occupancy rate = 0) from the northern parts of their current range within a few decades and likely to be extirpated (>50% probability) in many other portions of its range over a similar time frame. This study also corroborates the previous demographic meta‐analyses (Franklin et al., 2021) and suggests the potential for population collapse if proactive strategies that respond to known drivers are not adopted in the near term.

This analysis represents the first year of northern spotted owl monitoring using ARUs throughout the geographic range in the United States. Increasing years of data will enable the estimation of population trends (Lesmeister, Appel, et al., 2021). For example, passive acoustic monitoring had been ongoing for several years prior to 2023 on demographic study areas, allowing us to document a rapid decline in naïve occupancy within the Tyee demographic study area (Oregon Coast modeling region) from 38% in 2021 to 15% in 2023 (Lesmeister et al., 2024). These findings are an indicator of population distress and exemplify the value of this type of continuous monitoring. Investigating underlying causes, including barred owl encroachment and regional superabundance, habitat degradation, and altered prey availability, can help managers to develop strategies to prevent similar declines elsewhere (Wiens et al., 2021). Northern spotted owl population declines have been ongoing for decades (Franklin et al., 2021; Yackulic et al., 2019), but the rate of recent declines is troubling. By expanding monitoring efforts outside of historical demographic study areas, we increased the extent of the range sampled, and for the first time produced predictions of distribution across the entire northern spotted owl range within the United States.

Comparisons with historical and current estimates further contextualize the severity of contemporary declines. Pair occupancy rates for northern spotted owls reported here generally align with the trajectories documented in prior demography‐based studies (Franklin et al., 2021) despite differences in sampling units (hexagons vs. territories). Recent work has shown how call‐back and ARU‐based approaches can be integrated despite these differences in sampling units providing a path forward using integrated modeling approaches (Weldy et al., 2023). Demography‐based studies were restricted to inference within long‐term study areas, whereas the randomized design of this study enabled predictions across the entire US range of the northern spotted owl. This represents a major advancement in monitoring and conservation science because it provides a comprehensive perspective of variability in occupancy patterns and extinction risk. By expanding beyond historical study areas, we can now identify regions approaching functional extinction thresholds and prioritize management actions at scales that were previously unattainable. Although methodological differences influence absolute estimates, the consistent spatial and temporal trends observed across independent datasets strengthen the conclusion that large portions of the northern spotted owl's range are now approaching or have reached extirpation thresholds (Franklin et al., 2021; Noon & McKelvey, 1996; Ovaskainen & Hanski, 2003). Together, these findings underscore the urgency of proactive conservation strategies.

### Detection probabilities

Our most‐supported detection probability sub‐model included differences by modeling region, and the positive effect of the number of hours of recording per week and pair status. While our models incorporated key survey‐ and hexagon‐level covariates influencing detection probability, it may be that unmodeled heterogeneity in home range sizes or local vegetation structure contributed to observed differences in detection probabilities and latency to first detection across regions. Vegetation density and structure can strongly influence the effective recording radius of ARUs, altering the area sampled at each station (Yip et al., 2017). Future models could incorporate local home range sizes and high‐resolution vegetation structure data to better account for these hexagon‐specific differences in acoustic sampling effectiveness and to refine detection probability estimates accordingly.

The factors responsible for variation in detection probability from our models underscore how survey effort influences the accuracy of observed occupancy state classification, with implications for monitoring rare or declining species. The odds of detection were over 15 times higher for hexagons occupied by paired owls relative to those occupied by only single individuals due to increased male territorial vocalizations at pair‐occupied hexagons. Females remained especially elusive, comprising only 2.4% of confirmed four‐note territorial calls, reinforcing the value of accounting for imperfect detection and developing sex‐specific adjustments when interpreting pair occupancy and viability estimates. This pattern is consistent with detailed behavioral studies of California spotted owls (*Strix occidentalis occidentalis*) showing that breeding status and sex strongly influence vocalization rates, call types, and loudness, with breeding females producing very few territorial calls and calling primarily near the nest (Reid et al., 2022).

The status of territorial resident pairs better represents the overall population status than presence of adults since adults can persist for many years, but unpaired adults do not contribute to reproduction. Since pairs are more difficult to detect, accurate estimates of pair detectability can inform survey efforts for forest management projects throughout the Pacific Northwest. In this study, we found the probability of only detecting the male when the true state was occupied by a pair was high with few survey weeks, especially in regions of low occupancy rates, but additional weeks of surveys resulted in increased opportunity to also detect the female (Figure 2, bottom panel). In regions with higher use rates, such as the Klamath Mountains, the cumulative probability of detecting both individuals of a pair exceeded 0.8 after 6 weeks of surveys. In contrast, in low‐occupancy regions like the Washington Cascades and Oregon Coast Range, the probability of detecting the male and the female remained persistently low, rarely exceeding 0.6 despite extended survey effort. These results are consistent with previous findings of Appel et al. (2023) who found that in these regions a hexagon yielding only male detections after 6 weeks of surveys still had ~0.5 probability of supporting a pair. The detection of both sexes is likely influenced not only by behavioral factors, such as female vocalization rates, but also by underlying population condition. As species become rarer, the probability of observing the true occupancy state remains low even with substantial effort, a widely recognized challenge in conservation monitoring (Yoccoz et al., 2001). These findings reinforce the broader principle that, although survey effort improves inference, biological rarity and behavioral detection biases set inherent limits on our ability to observe the true biological state.

### Landscape use and pair occupancy

Occupancy modeling revealed nuanced interactions between latitude, topographic complexity, and forest composition. Landscape use and pair occupancy increased significantly with old‐growth structural complexity and topographic diversity, emphasizing the role of structurally complex habitats (Forsman et al., 1984). Pair occupancy demonstrated complex latitudinal interactions with hardwood canopy cover, potentially linked to higher prey availability, prey species diversity, and heterogeneity of forest types (Franklin et al., 2000), and lower barred owl landscape use rates in the southern regions (Franklin et al., 2021).

The predictive distribution maps that we generated in this study are intended to support regional‐scale conservation planning and prioritization efforts. The maps are not definitive landscape use and pair occupancy estimates at the scale of individual hexagons but the average likelihood that a given hexagon may be used by at least one northern spotted owl or occupied by a pair. Caution is warranted when interpreting fine‐scale predictions, particularly for rare species such as the northern spotted owl, where declining abundance and detectability increase prediction uncertainty. As a population declines, the ability to produce accurate spatial predictions at fine resolutions diminishes, a challenge well documented in rare species modeling (Cunningham & Lindenmayer, 2005; Jeliazkov et al., 2022; Zhang et al., 2020). This is particularly true when competitive interactions are driving the decline, as is the case with barred owls and spotted owls. Consequently, the maps of spotted owl distribution we produce can inform broader spatial patterns and highlight areas of relative conservation priority among stands or regions, rather than individual hexagons.

The pervasive presence of barred owls, confirmed in 87% of sampled hexagons at calling intensities eight times greater than northern spotted owls (Jenkins et al., 2025), confirms that barred owls remain a threat to northern spotted owl conservation and biodiversity across the range (Lesmeister et al., 2018; Wiens et al., 2021). Future research may consider using passive acoustic monitoring to better understand prey availability (e.g., Diggins et al., 2020) and competitive dynamics between these owls.

The apparent mismatch between northern spotted owl landscape use and pair occupancy with nesting and roosting forest availability (Figure 6) underscores substantial conservation and land management challenges. During the first quarter‐century of its implementation, the NWFP was largely successful in protecting and restoring forests suitable for northern spotted owl nesting and roosting (Davis et al., 2022; Lesmeister et al., 2018). However, despite the abundance of suitable nesting habitat in regions like the Oregon Coast Range, northern Klamath Mountains, and Washington Cascades, northern spotted owl pair occupancy remains alarmingly low. This disconnect likely results from intense barred owl competition where northern spotted owls are excluded from otherwise suitable habitat (Jenkins et al., 2025), underscoring the necessity of dual pronged approach to conservation actions for old forest and relieving competitive pressures (Wiens et al., 2021). Spatially targeted barred owl control has a narrow temporal window in which the method is more likely to be effective in lowering the probability of regional extirpations (Hofstadter et al., 2022).

Occupancy modeling showed strong associations between areas used by northern spotted owls and habitats characterized by forest structural complexity and topographic diversity, elements highly correlated at finer scales with fire refugia (Lesmeister, Davis, et al., 2021). Protecting these refugia through targeted management, wildfire risk mitigation, and restoration is likely to maintain biodiversity while safeguarding high quality spotted owl habitat (Lesmeister et al., 2025). This is especially relevant for our two southern regions that had the highest spotted owl occupancy rates and have the greatest wildfire risk (Lesmeister, Davis, et al., 2021). Such strategies are increasingly vital in fire‐prone ecosystems worldwide amid climate‐driven increases in wildfire frequency and severity.

Integrating large‐scale and long‐term barred owl control and fire refugia‐focused habitat conservation into adaptive, spatially explicit strategies may enhance northern spotted owl population persistence. Future work could build on our results by identifying conservation targets (e.g., pair occupancy rates), allowing managers to efficiently allocate resources to locations. By combining invasive species control, habitat preservation, and restoration within strategic frameworks informed by ongoing passive acoustic monitoring, managers can effectively address cumulative threats, enhance ecosystem resilience, and bolster long‐term viability of vulnerable wildlife populations.

## CONCLUSION

Our passive acoustic monitoring assessment corroborates findings by Franklin et al. (2021) that there is substantial extinction risk for northern spotted owls across much of their range, driven by invasive barred owls, habitat limitations, and challenges in detecting rare, behaviorally elusive individuals. Habitat protections for spotted owls can hinge on detecting both individuals of a pair, which is a substantial challenge for northern spotted owl females, especially when they are nesting. Despite the inherent challenges of spotted owl conservation, our results highlight the value of passive acoustic monitoring and machine learning as scalable, noninvasive tools for tracking population status at broad spatial and temporal scales. This approach offers advantages for wide‐ranging and low‐density species, where traditional methods are often logistically prohibitive. More broadly, biodiversity conservation faces mounting challenges from habitat degradation, invasive species, and shifting disturbance regimes (Cardinale et al., 2012; Dirzo et al., 2014). Conservation success depends on the ability to detect population changes early enough to guide meaningful intervention (Sugai & Llusia, 2019). Our findings underscore the importance of continued investment in innovative monitoring systems that can substantially enhance biodiversity monitoring, ecological forecasting, and adaptive conservation strategies.

## CONFLICT OF INTEREST STATEMENT

The authors declare no conflicts of interest.

## Supporting information


Appendix S1.

## Data Availability

Data and code (Jenkins et al., 2026) are available in Zenodo at https://doi.org/10.5281/zenodo.20275037.
