# Supplementary material for: Listening for extinction: Range‐wide occupancy highlights critical risks and priorities for northern spotted owls
Source: Ecol Appl. 2026 Aug 22;36(5):e70294. doi: 10.1002/eap.70294 (PMC13498988; doi:10.1002/eap.70294)
Supplement: Supplementary file 1 [file EAP-36-e70294-s001.pdf]

Appendix S1

Listening for extinction: Range-wide occupancy highlights critical risks and priorities for northern spotted owls

Damon B. Lesmeister, Julianna M. A. Jenkins, Raymond J. Davis, Natalie M. Rugg, Cara L. Appel, Larissa L. Bailey, Erin Bayne, Tara Chestnut, Leila S. Duchac, Katie M. Dugger, Taylor D. Ellis, Alan B. Franklin, Scott A. Gremel, Aaron Henderson, Edward Henson, J. Mark Higley, James E. Hines, Bruce Hollen, William L. Kendall, Taal Levi, Darryl I. MacKenzie, Christopher E. McCafferty, Thomas Munger, David T. Press, Caroline Provost, Suzanne Reffler, Zachary J. Ruff, James K. Swingle, Erica Tevini, Alaina D. Thomas, Matthew J. Weldy, Kirsten Wert, Angela White, Charles B. Yackulic

Ecological Applications

Multi-state, multi-season goodness-of-fit test.

The goodness-of-fit test used in this analysis follows the method of the single-season, single-state goodness-of-fit test proposed by MacKenzie and Bailey (2004). In the single-state case, occupancy and detection parameters are estimated for the desired model which are then used to generate many simulated datasets (specified by the user). Chi-square test statistics are computed for the original data and compared to the distribution of test statistics computed for the simulated datasets. The variance inflation factor, c-hat, was computed as the ratio of the observed data test statistic to the mean of the simulated data test statistics. The same concept is applied to the multi-state case, but occupancy parameters included both probability of landscape use by northern spotted owls regardless of pair state (ψ) and the probability that northern spotted owls were paired (female and male use), given that they were using a hexagon (R). Detection parameters included both state-specific detection probabilities (p) and probability of correctly assigning pair occupancy (δ).

R Code:

```
#rm(list=ls()); setwd('c:/users/…')
library(RPresence)
pao=readRDS('data/NSOpao.rds')
str(pao)
m1=readRDS('output/topMod1.rds')

#m1=x_mdls2[[8]]
m1$model$Cpsi=m1$model$CR=NULL
m1a=occMod(model=m1$model, data=pao, type=m1$type, modname=modname,
        outfile='topmod1a', modfitboot=1, threads=10, randinit=10)

sel.model=m1a

######################## GOF TEST #################################

gof <- function(fname) {
  tchi1=ndf1=tchi2=ndf2=tchi3=ndf3=0
```

```
  a=readLines(fname); i=grep('obs.i. ',a)    # i=line with "obs(i)" in output
  if (length(i)>0) {
    a1=gsub('-1','.',gsub(' +',' ',a[i+1:pao$nunits]))  # a1=lines with obs/exp vals
    a2=matrix(unlist(strsplit(a1,' ')),nrow=pao$nunits,byrow=T)
    obs=as.numeric(a2[,3]); exp=as.numeric(a2[,4])+1e-5
    df=data.frame(obs=obs, exp=exp, ch=a2[,1])
    chi=(obs-exp)^2/exp; tchi1=sum(chi); ndf1=nrow(df)-1

    #  try pooling detection histories which are the same...
    df=data.frame(ch=a2[,1], obs=obs, exp=exp, chi=round(chi,2))
    obs2=by(df$obs,df$ch,sum);   exp2=by(df$exp,df$ch,sum)
    s2=names(obs2)
    obs2=as.numeric(obs2); exp2=as.numeric(exp2)+1e-6
    chi2=(obs2-exp2)^2/exp2; tchi2=sum(chi2)
    df2=data.frame(ch=s2, obs=obs2, exp=exp2, chi=round(chi2,2))
    #print(df2,quote=F);
    ndf2=nrow(df2)-1
    #cat('  total chi-sq:',tchi2,' df:',ndf2,' prob:',1-pchisq(tchi2,ndf2),' c-hat:',tchi2/ndf2,'\n')

    #  try pooling more...
    df3=df2
    df3[4,-1]=colSums(df2[4:8,-1])
    df3[5:8,-1]=0
    chi3=(df3$obs-df3$exp)^2/df3$exp; tchi3=sum(chi3,na.rm=T)
    df3$chi=round(df3$chi,2); ndf3=sum(df3$exp>0)-1
    #print(df3,quote=F)
    #cat('  total chi-sq:',tchi3,' df:',ndf3,' prob:',1-pchisq(tchi3,ndf3),' c-hat:',tchi3/ndf3,'\n')
  }
  return(c(tchi1,ndf1,tchi2,ndf2,tchi3,ndf3))
}

N=pao$nunits; K=pao$nsurveys
psi=sel.model$real$psi[,1]; R=sel.model$real$R[,1]
p=array(
  c(rep(0,N*K),                                         # p(state=1 : not occ)
    sel.model$real$p[grep('p1',rownames(sel.model$real$p)),1], # p(state=2 : occ)
    sel.model$real$p[grep('p2',rownames(sel.model$real$p)),1]  # p(state=3 : breed)
  ), dim=c(N, K, 3), dimnames=NULL
)
delta=array(
  c(rep(0,N*K),                  # delta(state1)=0  not occ
    rep(0,N*K),                  # delta(state2)=0  no breeding
    sel.model$real$delta[,1]),   # delta(state3)=delta  breeding
  dim=c(N,K,3),dimnames=NULL
)
psi[is.na(psi)]=R[is.na(R)]=p[is.na(p)]=delta[is.na(delta)]=0
```

```
delta[,3,3]=0   # last delta fixed to zero in model

nsims=100; m1=list(); orig_det.data=pao$det.data; dh=matrix(0,N,K)
tbar=txtProgressBar(max=nsims, width=50, style=3)

sel.model$model$Cpsi=sel.model$model$CR=NULL
cat('orig data: nll=',sel.model$neg2loglike,'\n');
gg=c(sel.model$neg2loglike,mean(sel.model$real$psi[,1]), gof(sel.model$outfile))

t1=Sys.time(); print(t1)
for (isim in 1:nsims) {
  setTxtProgressBar(tbar, isim)
  occ=0+(runif(N)<psi)     # 0=unocc, 1=occ
  brd=0+(runif(N)<R)       # 0=not breeding, 1=breeding
  state=1 + occ + occ*brd  # 1=unocc, 2=occ w/o breeding, 3=occ w/ Breeding

  for (i in 1:K) {
    mi=cbind(1:N,i,state)     # subscripts for p,delta
    d1=0+(runif(N)<p[mi])     # di=0: not detected, 1:detected
    #  note: if state=1, p=0
    d2=0+(runif(N)<delta[mi]) # d2=2: detected w/ breeding
    #   note: if state=1 or 2, delta=0
    dh[,i]=d1+d2
  }
  dh[is.na(orig_det.data)]
  dh[is.na(pao$det.data)]=NA

  pao$det.data=dh

  m1[[isim]]=occMod(model=sel.model$model, data=pao, type=sel.model$type,
                  modfitboot=1, quiet=T, threads=10, outfile='sim.out')

  g=c(m1[[isim]]$neg2loglike,mean(m1[[isim]]$real$psi[,1]),gof(m1[[isim]]$outfile))

  gg=rbind(gg,g)
}
close(tbar); t2=Sys.time(); print(t2)
colnames(gg)=c('neg2ll','psi','chi1','df1','chi2','df2','chi3','df3')
rownames(gg)=c('orig.data',paste0('sim',1:nsims))
print(gg)

cat('c-hat:',gg[1,3]/mean(gg[-1,3]),'\n')
c.hat <- gg[1,3]/mean(gg[-1,3])
cat('prob(chi-data|chi-simulated):',sum(gg[1,3]<gg[-1,3])/nsims,'\n')
cat('cpu time:\n')
print(difftime(t2,t1))
```

Out of sample predictions.

We compared the area sampled with the total area for application. We initially selected our random sample from all 5-km^2 hexagons which were ≥25% federally managed and ≥50% forest capable. We then explored the range of conditions within our ‘sample’ compared to all federally managed forest capable lands (area available for sampling), and across all forested lands and found that our area sampled had similar range of conditions for the variables in our top models for all forested lands (Fig. S1, Table S1). From the most-supported model of occupancy [ψ(LAT + TOPODIV + OGSImn)R(LAT×CCOV_HDW + OGSImn)], we compared the mean probabilities of derived occupancy parameters, northern spotted owl landscape use (ψ), use by a single owl (ϕ1), and pair occupancy (ϕ2). We found the differences were minor amongst our sample and all forested lands with a few exceptions (Fig. S2). The California Coast (CACOA) and Washington Coast and Western Cascades (WWOLY) had disproportionate amounts of non-federal lands, particularly in lowland areas, where northern spotted owl nesting and roosting was not available.

REFERENCE

MacKenzie, D. I., and L. L. Bailey. 2004. Assessing the fit of site-occupancy models. Journal of Agricultural, Biological, and Environmental Statistics **9**:300-318.

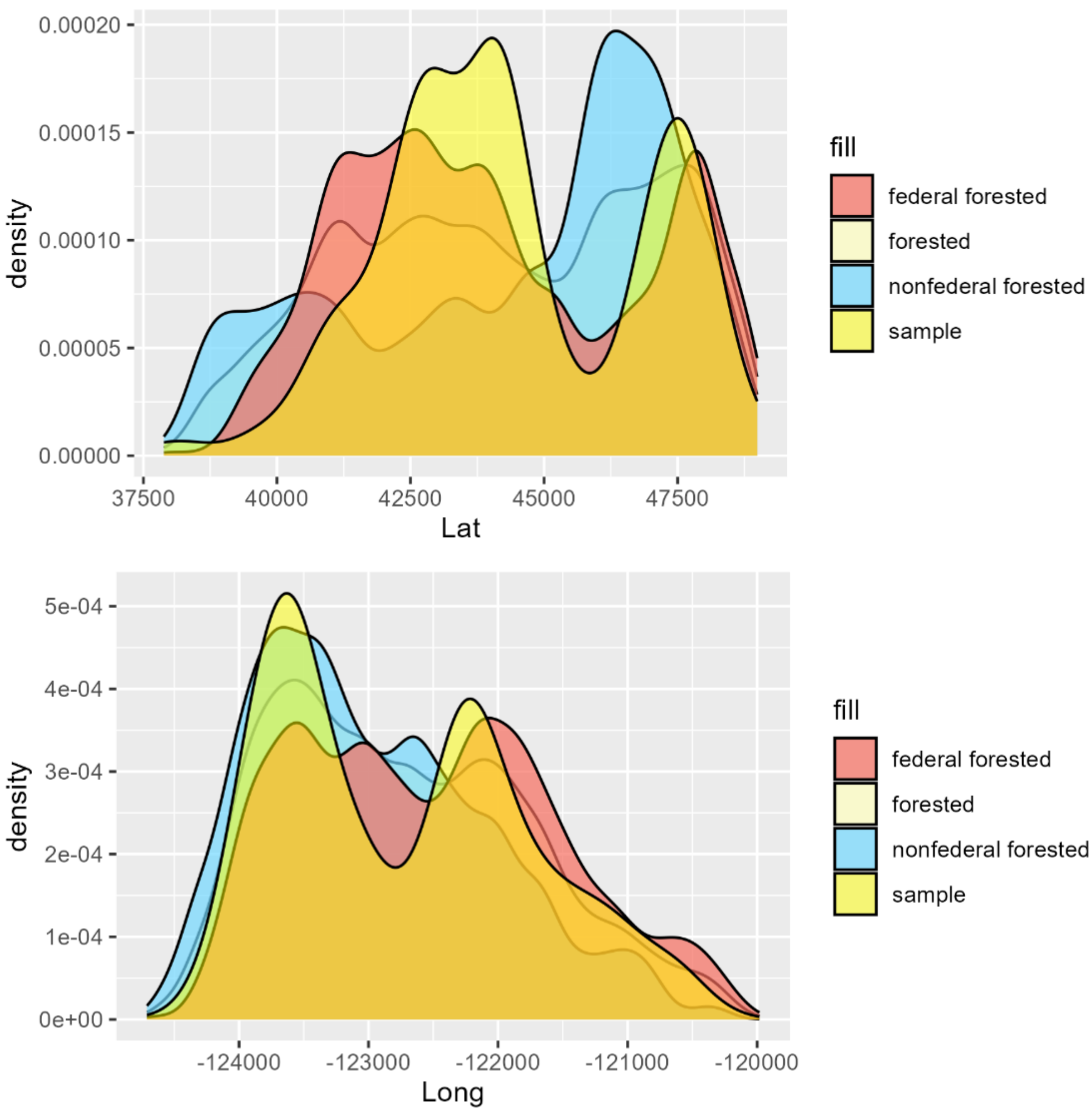

Figure S1. Geographic distribution of sampled hexagons relative to the full extent of forested lands across the northern spotted owl range. The density plots demonstrate how our randomly selected 5-km² sampled hexagons, which were restricted to areas ≥25% federally managed and ≥50% forest capable, are spatially representative of all forested lands, federally managed forest-capable lands, and nonfederal forested lands. The sampled area encompassed the full range of environmental conditions included in the most-supported multistate occupancy model, indicating that inference from the fitted model is broadly applicable across the range.

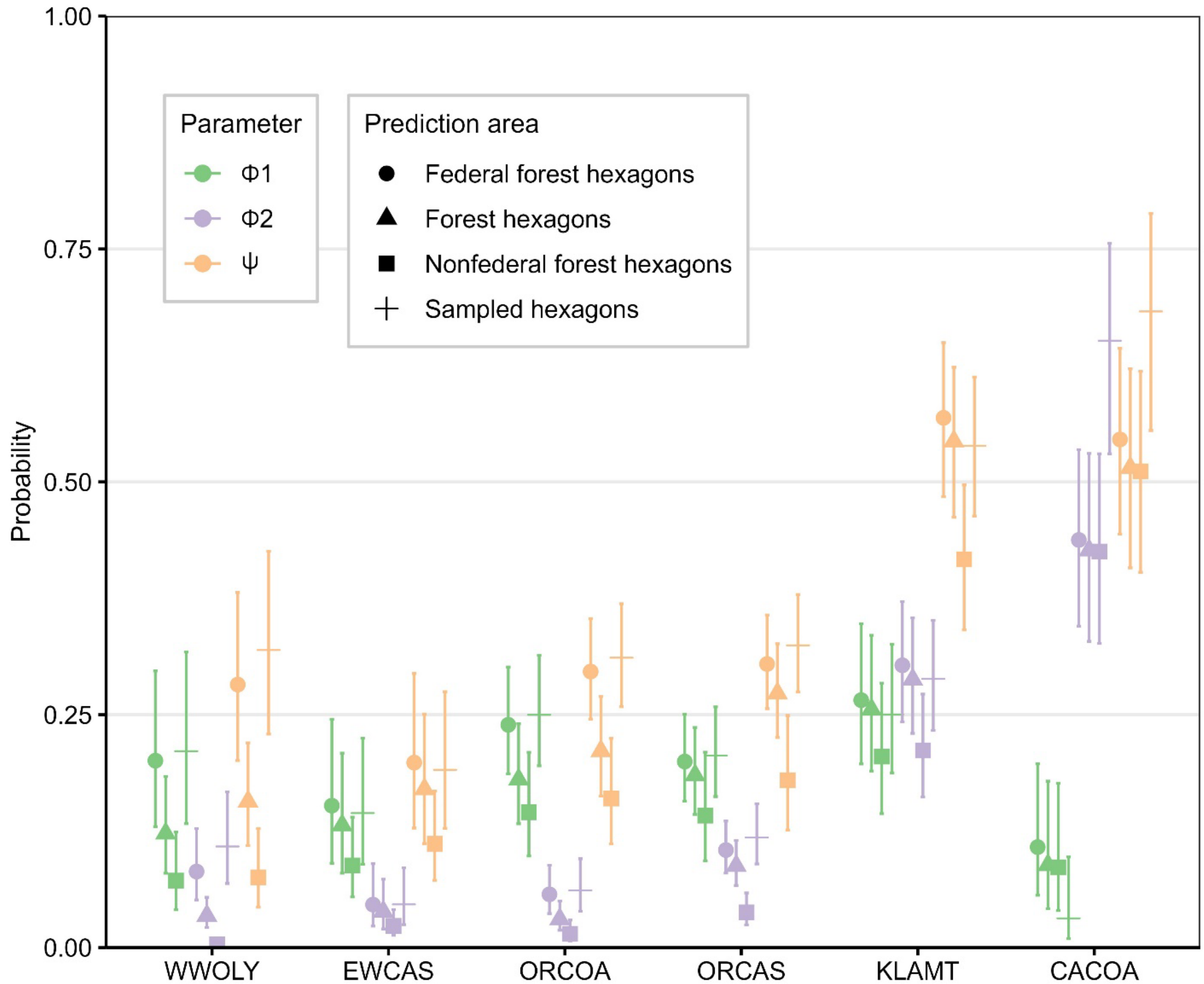

Figure S2. Mean derived occupancy values for northern spotted owl landscape use (ψ), use by a single owl (ϕ1), and pair occupancy (ϕ2) with 95% confidence intervals overlapped forsampled, forested, and federal forest prediction areas for all but pair occupancy in the CACOA where our sampled hexagons had higher predicted pair occupancy than other prediction areas. Generally, federal forest lands encompassed higher mean occupancy values compared to nonfederal forest, which aligns with the distribution of northern spotted owl nesting and roosting forest, concentrated in areas under federal management. Modeling regions: KLAMT = Oregon and California Klamath Mountains; CACOA = California Coast; EWCAS = Washington Eastern Cascades; ORCAS = Oregon and California Cascades; ORCOA = Oregon Coast Range; WWOLY = Washington Coast and Western Cascades.

Table S1. Summary statistics for covariates included in the 2023 multistate occupancy model using passive acoustic monitoring data of northern spotted owls. Mean and standard deviation values for the rangewide sample was used when scaling data.

	Rangewide hexagons ≥ 50% forest capable (n = 38,341)				Sampled Hexagons (n = 1,027)			
Covariate	Mean	SD	Min	Max	Mean	SD	Min	Max
FRmn	28	11	0	66	29	10	2	61
FRp	37	29	0	100	38	29	0	100
NRp	23	23	0	99	40	23	0	99
NR_INT	42	68	0	486	83	84	0	478
CLIMWD	377	219	0	1221	365	193	26	985
TPI270	-1.2	208	-1219	1180	-6.4	225	-1053	689
CHILI	172	19	60	226	171	19	79	225
TOPODIV	63	23	5	100	69	19	12	100
ELEVmn	763	503	4	2285	894	481	29	2135
ELEVsd	101	59	1	429	113	57	6	406
CC_HDW	17	15	0	80	12	14	0	70
OGSImn	29	11	0	77	36	9.7	5	70
OGSI80	411	232	2	994	578	199	12	984
LAT	44264	2816	37883	48991	44417	2373	37894	48945

Table S2. Survey covariate correlations using Spearman's rank. Correlations >0.60 are in bold. See Table 1 for covariate descriptions.

	DATE	SPL	PPRCP	PPRCPmn	TPI270	ELEVsd	TEMPmn	TEMPmx	TOPODIV	ln(effort)	LAT	BO-PC1
DATE	**1.00**											
SPL	-0.29	**1.00**										
PPRCP	-0.53	0.27	**1.00**									
PPRCPmn	-0.53	0.27	**1.00**	**1.00**								
TPI270	0.05	-0.34	-0.06	-0.06	**1.00**							
ELEVsd	0.31	0.26	-0.13	-0.13	-0.05	**1.00**						
TEMPmn	**0.72**	-0.33	**-0.68**	**-0.68**	0.10	0.20	**1.00**					
TEMPmx	**0.65**	-0.33	**-0.71**	**-0.71**	0.10	0.14	**0.98**	**1.00**				
TOPODIV	0.27	0.26	-0.10	-0.10	-0.14	**0.87**	0.19	0.12	**1.00**			
ln(effort)	-0.07	-0.03	0.05	0.04	0.03	-0.06	-0.03	-0.02	-0.04	**1.00**		
LAT	0.10	0.24	0.07	0.07	-0.11	0.35	-0.15	-0.22	0.37	0.03	**1.00**	
BO-PC1	-0.04	-0.38	-0.04	-0.04	0.10	-0.12	0.01	0.01	-0.05	0.19	0.14	**1.00**

Table S3. Hexagon covariate correlations using Spearman's rank. Correlations >0.60 are in bold. See Table 1 for covariate descriptions.

	LAT	FRmn	FRp	NR_ INT	NRp	CLIM WD	TPI 270	CHILI	TOPO DIV	ELEV mn	ELEV sd	CCOV _HDW	OGSI mn	OGSI 80	FOR EST-PC1	BO time	BO-PC1
LAT	**1.00**																
FRmn	0.13	**1.00**															
FRp	0.20	**0.96**	**1.00**														
NR_INT	0.10	0.05	0.02	**1.00**													
NRp	0.10	0.11	0.08	**0.95**	**1.00**												
CLIMWD	**-0.72**	-0.01	-0.08	-0.04	-0.04	**1.00**											
TPI270	-0.11	-0.24	-0.29	-0.09	-0.08	0.01	**1.00**										
CHILI	-0.43	-0.07	-0.11	-0.09	-0.11	0.24	-0.01	**1.00**									
TOPODIV	0.36	0.08	0.15	0.30	0.28	-0.18	-0.13	-0.40	**1.00**								
ELEVmn	-0.14	**-0.71**	**-0.73**	-0.13	-0.19	-0.10	0.10	0.10	-0.02	**1.00**							
ELEVsd	0.34	-0.07	-0.01	0.27	0.24	-0.22	-0.04	-0.31	**0.88**	0.13	**1.00**						
CC_HDW	-0.38	0.47	0.46	0.11	0.15	0.53	0.03	0.07	0.02	**-0.70**	-0.09	**1.00**					
OGSImn	0.00	0.08	0.04	0.58	**0.66**	-0.15	0.07	-0.02	0.13	-0.01	0.13	0.02	**1.00**				
OGSI80	0.04	-0.07	-0.11	0.51	0.59	-0.20	0.03	0.00	0.05	0.22	0.08	-0.22	**0.86**	**1.00**			
FOREST-PC1	0.30	0.23	0.21	**0.63**	**0.71**	-0.43	0.01	-0.10	0.14	-0.16	0.12	-0.10	**0.79**	**0.72**	**1.00**		
BOtime	**0.83**	0.18	0.25	-0.03	0.00	**-0.61**	-0.06	-0.38	0.32	-0.12	0.29	-0.27	-0.06	0.02	0.18	**1.00**	
BO-PC1	0.17	0.28	0.26	0.28	0.30	-0.17	0.12	-0.11	-0.05	-0.35	-0.11	0.17	0.19	0.10	0.38	0.13	**1.00**

Table S4. Sampling and naïve occupancy summaries. For each modeling region, the number of all forest capable hexagons (on federal and nonfederal lands), forest capable hexagons with ≥25% federal land, number of hexagons surveyed, and percentage of available hexagons surveyed. Also reported are the number of hexagons surveyed with highest observed state of northern spotted owls (state 0 = no detections, state 1 = single owl detected, state 2 = pair detected) and with barred owl detections. The spotted owl and barred owl naïve occupancy we report is the percentage of surveyed hexagons with at least one confirmed detection of northern spotted owl or barred owl, respectively. Modeling regions: California Coast (CACOA); Oregon and California Klamath Mountains (KLAMT); Oregon Coast Range (ORCOA); Oregon and California Cascades (ORCAS); Washington Eastern Cascades (EWCAS); and Washington Coast and Western Cascades (WWOLY).

	Modeling region						
	CACOA	KLAMT	ORCOA	ORCAS	EWCAS	WWOLY	Total
Forest capable hexagons	3,337	7,931	5,045	8,066	3,479	10,483	38,341
Federal forest capable hexagons	379	6,606	1,890	6,032	2,344	4,127	21,378
Percent of forest capable hexagons with >25% federal	11.4%	83.3%	37.5%	74.8%	67.4%	39.4%	55.8%
Sampled hexagons	11	191	208	300	105	212	1,027
Percent of federal forested hexagons surveyed	2.9%	2.9%	11.0%	5.0%	4.5%	5.1%	4.8%
Percent of all forested hexagons surveyed	0.3%	2.4%	4.1%	3.7%	3.0%	2.0%	2.7%
Observed state 0	1	107	182	213	87	176	766
Observed state 1	0	43	24	59	16	30	172
Observed state 2	10	41	2	28	2	6	89
Spotted owl naïve occupancy	90.9%	44.0%	12.5%	29.0%	17.1%	17.0%	25.4%
Barred owl detections	5	137	207	257	86	199	891
Barred owl naïve occupancy	45.5%	71.7%	99.5%	85.7%	81.9%	93.9%	86.8%

Table S5. Top ranked sub-models ordered by Quasi Akaike Information Criterion (QAIC) values and model weights (*w*). We fit models to evaluate covariates affecting state-specific detection probability (*p*), detection or correct classification of pairs (δ), landscape use probability (ψ), and the probability of pairing given use (*R*) of northern spotted owls throughout their range in 2023. Reported are the number of parameters (*K*) and the non-focal parameter structure used for each sub-model set is given in backets, [ ]. For the parameter *R*, we combined the regions Oregon and California Klamath Mountains and California Coast (MR5), which was hindered due to small sample size and the high rate of R (near 1.00). See Table 1 for covariate descriptions. Models with interactions also contain their additive parts.

Model	QAIC	*K*	ΔQAIC	*w*
Detection sub-models: [ψ(MR)*R*(MR5)δ(MR)]				
p(STATE+ln(effort)+MR)*	3419.10	25	0.00	0.86
p(STATE+ln(effort)+ ARU:MR)	3422.79	27	3.69	0.14
p(STATE+ln(effort)+PPRCPmn)	3446.22	21	27.12	0.00
p(STATE+ln(effort)+PPRCP)	3446.63	21	27.54	0.00
p(STATE+ln(effort)+PPRCPmn+SPL	3447.70	22	28.60	0.00
p(STATE+ln(effort)+TEMPmx)	3452.91	21	33.81	0.00
p(STATE+ln(effort)+TEMPmn)	3453.55	21	34.45	0.00
p(STATE+ln(effort)+SPL)	3457.20	21	38.10	0.00
p(STATE+ln(effort))	3458.61	20	39.51	0.00
p(STATE+ln(effort)+DATE)	3459.02	21	39.92	0.00
p(STATE+ln(effort)+ELEVsd)	3459.17	21	40.08	0.00
p(STATE+ln(effort)+BO)	3459.17	21	40.08	0.00
p(STATE+ln(effort)+ARU)	3460.20	21	41.10	0.00
p(STATE+ln(effort)+TPI270)	3460.44	21	41.35	0.00
p(STATE+ln(effort)+TOPODIV)	3460.47	21	41.38	0.00
p(STATE+ln(effort)+BO-PC1)	3460.54	21	41.44	0.00
p(STATE+ln(effort)+DATE+ $DATE^2$)	3461.02	22	41.92	0.00
Correct Classification sub-models: [ψ(MR)*R*(MR5)*p*(STATE+ln(effort)+MR)]				
δ(MR+TPI270)*	3411.18	26	0.00	0.76
δ(MR+PPRCP)*	3415.08	26	3.90	0.11
δ(MR+BO)*	3418.53	26	7.35	0.02
δ(MR)*	3419.10	25	7.92	0.01
δ(MR×ELEVsd)	3419.98	31	8.80	0.01
δ(MR+DATE)	3420.96	26	9.78	0.01
δ(MR+ELEVsd)	3420.99	26	9.81	0.01
δ(MR×DATE)	3426.11	31	14.93	0.00
δ(LAT+TPI270)	3430.63	22	19.45	0.00
δ(LAT×TPI270)	3432.59	23	21.41	0.00
δ(LAT×DATE)	3435.70	23	24.52	0.00

δ(LAT×BO)	3436.10	23	24.92	0.00
δ(LAT+PPRCP)	3437.15	22	25.97	0.00
δ(LAT+BO)	3437.53	22	26.35	0.00
δ(LAT+PPRCP+DATE)	3437.60	23	26.42	0.00
δ(LAT)	3438.85	21	27.67	0.00
δ(LAT×PPRCP)	3438.99	23	27.81	0.00
δ(LAT×ELEVsd)	3440.39	23	29.21	0.00
δ(LAT+DATE)	3440.83	22	29.65	0.00
δ(LAT+ELEVsd)	3440.85	22	29.67	0.00
δ(MR5)	3456.38	24	45.20	0.00
δ(MR5+BO)	3457.98	25	46.80	0.00
δ(MR5×BO)	3465.07	29	53.89	0.00
δ(TPI270)	3482.24	21	71.06	0.00
δ(ELEVsd)	3483.32	21	72.14	0.00
δ(DATE)	3483.90	21	72.72	0.00
δ(DATE+DATE^2)	3485.80	22	74.62	0.00
δ(PPRCP)	3486.60	21	75.43	0.00
δ(PPRCPmn)	3486.83	21	75.65	0.00
δ(TOPODIV)	3488.15	21	76.97	0.00
δ(BO)	3488.15	21	76.97	0.00
δ(SPL)	3488.34	21	77.16	0.00
δ(Intercept)	3488.39	20	77.21	0.00
δ(TEMPmx)	3489.10	21	77.92	0.00
δ(BO-PC1)	3489.70	21	78.53	0.00
δ(TEMPmn)	3489.73	21	78.55	0.00
δ(ln(effort))	3490.37	21	79.19	0.00
Landscape use sub-models: [*R*(MR5)*p*(STATE+ln(effort)+MR)δ(MR)]				
ψ(TOPODIV+OGSImn+LAT)*	3367.07	23	0.00	0.97
ψ(TOPODIV+OGSImn+BOtime)*	3375.06	23	7.99	0.02
ψ(MR×TOPODIV+OGSImn)	3378.10	32	11.03	0.00
ψ(MR×TOPODIV+OGSImn+CLIMWD)	3379.21	33	12.14	0.00
ψ(MR+TOPODIV+OGSImn)	3379.94	27	12.87	0.00
ψ(MR+TOPODIV+OGSImn+CLIMWD)	3380.66	28	13.60	0.00
ψ(TOPODIV+LAT)	3383.31	22	16.25	0.00
ψ(TOPODIV×LAT)	3384.44	23	17.37	0.00
ψ(LAT×TOPODIV+CC_HDW)	3386.03	24	18.97	0.00
ψ(TOPODIV×BOtime)	3386.89	23	19.82	0.00
ψ(TOPODIV+BOtime)	3387.22	22	20.15	0.00
ψ(TOPODIV+OGSImn)	3389.96	22	22.89	0.00

ψ(TOPODIV+OGSImn+BO-PC1)	3390.30	23	23.23	0.00
ψ(TOPODIV×OGSImn)	3391.37	23	24.30	0.00
ψ(OGSImn+LAT)	3393.33	22	26.26	0.00
ψ(TOPODIV+MR)	3395.66	26	28.60	0.00
ψ(MR×TOPODIV+CLIMWD)	3396.09	32	29.02	0.00
ψ(OGSImn+BOtime)	3397.75	22	30.68	0.00
ψ(OGSImn+MR)	3399.95	26	32.88	0.00
ψ(TOPODIV+BO-PC1)	3403.64	22	36.57	0.00
ψ(TOPODIV)	3404.66	21	37.59	0.00
ψ(OGSImn+BO-PC1)	3406.82	22	39.75	0.00
ψ(OGSImn)	3406.96	21	39.89	0.00
ψ(LAT+BO-PC1)	3411.12	22	44.05	0.00
ψ(NRp)	3414.52	21	47.45	0.00
ψ(BOtime+BO-PC1)	3414.57	22	47.50	0.00
ψ(LAT)	3416.68	21	49.61	0.00
ψ(BOtime)	3417.27	21	50.20	0.00
ψ(FOREST-PC1)	3417.89	21	50.83	0.00
ψ(MR)	3419.10	25	52.03	0.00
ψ(CC_HDW)	3419.32	21	52.26	0.00
ψ(ELEVsd)	3419.44	21	52.38	0.00
ψ(NR_INT)	3422.73	21	55.66	0.00
ψ(OGSI80p)	3424.78	21	57.71	0.00
ψ(CLIMWD)	3425.01	21	57.94	0.00
ψ(BO-PC1)	3426.20	21	59.13	0.00
ψ(Intercept)	3428.30	20	61.24	0.00
ψ(TPI270mn)	3428.47	21	61.40	0.00
ψ(CHILI)	3429.46	21	62.39	0.00
ψ(ELEVmn)	3429.60	21	62.53	0.00
ψ(FRp)	3429.97	21	62.90	0.00
ψ(FRmn)	3430.08	21	63.02	0.00
ψ(BO)	3430.28	21	63.21	0.00
Probability of pairing given use:[ψ(MR)*p*(STATE+ln(effort)+MR)δ(MR)]				
R(LAT+ELEVsd+OGSImn)*	3383.66	24	0.00	0.51
R(LAT×CC_HDW+OGSImn)*	3383.86	25	0.20	0.46
R(BOtime+ELEVsd+OGSImn)*	3390.47	24	6.81	0.02
R(LAT×CC_HDW+ELEVsd)*	3391.87	25	8.21	0.01
R(ELEVsd+OGSImn+CLIMWD)	3394.41	24	10.75	0.00
R(BOtime+ELEVsd)	3397.18	23	13.52	0.00
R(LAT×ELEVsd)	3397.69	24	14.03	0.00

R(LAT+ELEVsd)	3398.24	23	14.58	0.00
R(BOtime×ELEVsd)	3399.10	24	15.44	0.00
R(OGSImn+LAT)	3400.09	23	16.43	0.00
R(OGSImn×LAT)	3400.51	24	16.85	0.00
R(LAT×CC_HDW)	3401.21	24	17.55	0.00
R(OGSImn+CLIMWD)	3401.57	23	17.91	0.00
R(OGSImn×CLIMWD)	3402.53	24	18.87	0.00
R(BOtime+OGSImn)	3403.18	23	19.52	0.00
R(BOtime×OGSImn)	3405.18	24	21.52	0.00
R(OGSImn+MR5)	3405.94	26	22.28	0.00
R(OGSImn+CC_HDW)	3405.97	23	22.31	0.00
R(MR5×CLIMWD)	3409.00	30	25.35	0.00
R(MR5×CLIMWD)	3409.00	30	25.35	0.00
R(OGSImn×MR5)	3409.31	30	25.65	0.00
R(OGSImn+ELEVsd)	3411.74	23	28.08	0.00
R(OGSImn×ELEVsd)	3412.24	24	28.58	0.00
R(BOtime+CLIMWD)	3414.33	23	30.67	0.00
R(BOtime+CC_HDW)	3414.38	23	30.72	0.00
R(BOtime)	3415.06	22	31.40	0.00
R(LAT×CLIMWD)	3415.40	24	31.74	0.00
R(OGSImn)	3416.53	22	32.87	0.00
R(LAT)	3417.10	22	33.44	0.00
R(MR5×CC_HDW)	3417.87	30	34.21	0.00
R(LAT+CC_HDW)	3417.92	23	34.26	0.00
R(LAT+CLIMWD)	3418.62	23	34.96	0.00
R(MR5)	3419.10	25	35.44	0.00
R(MR5+CC_HDW)	3420.61	26	36.95	0.00
R(ELEVsd)	3421.28	22	37.62	0.00
R(CC_HDW)	3421.58	22	37.92	0.00
R(CLIMWD)	3421.86	22	38.20	0.00
R(TOPODIV)	3422.23	22	38.57	0.00
R(OGSI80)	3425.82	22	42.16	0.00
R(FRp)	3428.76	22	45.10	0.00
R(Intercept)	3428.93	21	45.27	0.00
R(BO)	3429.26	22	45.61	0.00
R(FOREST-PC1)	3429.36	22	45.70	0.00
R(NRp)	3429.54	22	45.88	0.00
R(FRmn)	3429.78	22	46.12	0.00
R(BO-PC1)	3430.14	22	46.48	0.00
R(ELEVmn)	3430.84	22	47.18	0.00

R(CHILI)	3430.88	22	47.22	0.00
R(TPI270)	3430.90	22	47.24	0.00
R(NR_INT)	3430.92	22	47.27	0.00

* These models were carried forward in subsequent stages and included in the final model set (Tables 2 and 3).

Table S6. Final set of top-ranked models ordered by Quasi Akaike Information Criterion (QAIC) values and model weights (*w*). We fit models to evaluate covariates affecting state-specific detection probability (p), detection or correct classification of pairs (δ), landscape use probability (ψ), and the probability of pairing given use (R) of northern spotted owls throughout their range in 2023. Reported are the number of parameters (*K*) and distance from lowest QAIC (ΔQAIC). See table 1 for covariate descriptions. Models with interactions also contain their additive parts.

Model	QAIC	*K*	ΔQAIC	*w*
ψ(LAT+TOPODIV+OGSImn)*R*(LAT×CC_HDW+ OGSImn)*p*(STATE+ln(effort)+MR)δ(MR+TPI270)	3337.30	24	0.00	0.48
ψ(LAT+TOPODIV+OGSImn)*R*(LAT+ELEVsd+OGSImn) *p*(STATE+ln(effort)+MR)δ(MR+TPI270)	3339.56	23	2.26	0.16
ψ(LAT+TOPODIV+OGSImn)*R*(LAT×CC_HDW+ ELEVsd)*p*(STATE+ln(effort)+MR)δ(MR+TPI270)	3339.87	24	2.56	0.13
ψ(LAT+TOPODIV+OGSImn)*R*(BOtime+ELEVsd+ OGSImn)*p*(STATE+ln(effort)+MR)δ(MR+TPI270)	3341.23	23	3.92	0.07
ψ(LAT+TOPODIV+OGSImn)*R*(LAT×CC_HDW+ OGSImn)*p*(STATE+ln(effort)+MR)δ(MR+PPRCP)	3341.95	24	4.64	0.05
ψ(LAT+TOPODIV+OGSImn)*R*(LAT×CC_HDW+ ELEVsd)*p*(STATE+ln(effort)+MR)δ(MR+PPRCP)	3343.88	24	6.58	0.02
ψ(TOPODIV+OGSImn+BOtime)*R*(LAT×CC_HDW+ OGSImn)*p*(STATE+ln(effort)+MR)δ(MR+TPI270)	3343.91	24	6.60	0.02
ψ(LAT+TOPODIV+OGSImn)*R*(LAT+ELEVsd+OGSImn) *p*(STATE+ln(effort)+MR)δ(MR+PPRCP)	3344.02	23	6.71	0.02
ψ(TOPODIV+OGSImn+BOtime)*R*(LAT+ELEVsd+ OGSImn)*p*(STATE+ln(effort)+MR)δ(MR+TPI270)	3344.65	23	7.34	0.01
ψ(LAT+TOPODIV+OGSImn)*R*(LAT×CC_HDW+ OGSImn)*p*(STATE+ln(effort)+MR)δ(MR+BO)	3344.89	24	7.59	0.01
ψ(LAT+TOPODIV+OGSImn)*R*(LAT×CC_HDW+ OGSImn)*p*(STATE+ln(effort)+MR)δ(MR)	3345.39	23	8.08	0.01
ψ(LAT+TOPODIV+OGSImn)*R*(BOtime+ELEVsd+ OGSImn)*p*(STATE+ln(effort)+MR)δ(MR+PPRCP)	3345.95	23	8.64	0.01
ψ(TOPODIV+OGSImn+BOtime)*R*(LAT×CC_HDW+ ELEVsd)*p*(STATE+ln(effort)+MR)δ(MR+TPI270)	3346.17	24	8.87	0.01
ψ(LAT+TOPODIV+OGSImn)*R*(LAT×CC_HDW+ ELEVsd)*p*(STATE+ln(effort)+MR)δ(MR+BO)	3346.56	24	9.26	0.00
ψ(LAT+TOPODIV+OGSImn)*R*(LAT+ELEVsd+OGSImn) *p*(STATE+ln(effort)+MR)δ(MR+BO)	3347.20	23	9.89	0.00
ψ(LAT+TOPODIV+OGSImn)*R*(LAT×CC_HDW+ ELEVsd)*p*(STATE+ln(effort)+MR)δ(MR)	3347.30	23	10.00	0.00
ψ(LAT+TOPODIV+OGSImn)*R*(LAT+ELEVsd+OGSImn) *p*(STATE+ln(effort)+MR)δ(MR)	3347.73	22	10.43	0.00
ψ(TOPODIV+OGSImn+BOtime)*R*(LAT×CC_HDW+ OGSImn)*p*(STATE+ln(effort)+MR)δ(MR+PPRCP)	3348.44	24	11.14	0.00
ψ(TOPODIV+OGSImn+BOtime)*R*(LAT+ELEVsd+ OGSImn)*p*(STATE+ln(effort)+MR)δ(MR+PPRCP)	3349.05	23	11.74	0.00
ψ(LAT+TOPODIV+OGSImn)*R*(BOtime+ELEVsd+	3349.63	23	12.32	0.00

OGSImn)*p*(STATE+ln(effort)+MR)δ(MR+BO)				
ψ(LAT+TOPODIV+OGSImn)*R*(BOtime+ELEVsd+ OGSImn)*p*(STATE+ln(effort)+MR)δ(MR)	3350.05	22	12.74	0.00
ψ(TOPODIV+OGSImn+BOtime)*R*(LAT×CC_HDW+ ELEVsd)*p*(STATE+ln(effort)+MR)δ(MR+PPRCP)	3350.15	24	12.84	0.00
ψ(TOPODIV+OGSImn+BOtime)*R*(LAT×CC_HDW+ OGSImn)*p*(STATE+ln(effort)+MR)δ(MR+BO)	3351.40	24	14.10	0.00
ψ(TOPODIV+OGSImn+BOtime)*R*(BOtime+ELEVsd+ OGSImn)*p*(STATE+ln(effort)+MR)δ(MR+TPI270)	3351.66	23	14.35	0.00
ψ(TOPODIV+OGSImn+BOtime)*R*(LAT×CC_HDW+ OGSImn)*p*(STATE+ln(effort)+MR)δ(MR)	3351.89	23	14.59	0.00
ψ(TOPODIV+OGSImn+BOtime)*R*(LAT+ELEVsd+ OGSImn)*p*(STATE+ln(effort)+MR)δ(MR+BO)	3352.24	23	14.94	0.00
ψ(TOPODIV+OGSImn+BOtime)*R*(LAT+ELEVsd+ OGSImn)*p*(STATE+ln(effort)+MR)δ(MR)	3352.78	22	15.47	0.00
ψ(TOPODIV+OGSImn+BOtime)*R*(LAT×CC_HDW+ ELEVsd)*p*(STATE+ln(effort)+MR)δ(MR+BO)	3352.85	24	15.54	0.00
ψ(TOPODIV+OGSImn+BOtime)*R*(LAT×CC_HDW+ ELEVsd)*p*(STATE+ln(effort)+MR)δ(MR)	3353.57	23	16.27	0.00
ψ(TOPODIV+OGSImn+BOtime)*R*(BOtime+ELEVsd+ OGSImn)*p*(STATE+ln(effort)+MR)δ(MR+PPRCP)	3356.70	23	19.39	0.00
ψ(TOPODIV+OGSImn+BOtime)*R*(BOtime+ELEVsd+ OGSImn)*p*(STATE+ln(effort)+MR)δ(MR+BO)	3360.43	23	23.13	0.00
ψ(TOPODIV+OGSImn+BOtime)*R*(BOtime+ELEVsd+ OGSImn)*p*(STATE+ln(effort)+MR)δ(MR)	3360.80	22	23.50	0.00
